# Contribution of Cerebellar Sensorimotor Adaptation to Hippocampal Spatial Memory

**DOI:** 10.1371/journal.pone.0032560

**Published:** 2012-04-02

**Authors:** Jean-Baptiste Passot, Denis Sheynikhovich, Éléonore Duvelle, Angelo Arleo

**Affiliations:** Laboratory of Neurobiology of Adaptive Processes, UMR 7102, CNRS – University Pierre and Marie Curie, Paris, France; The University of Plymouth, United Kingdom

## Abstract

Complementing its primary role in motor control, cerebellar learning has also a bottom-up influence on cognitive functions, where high-level representations build up from elementary sensorimotor memories. In this paper we examine the cerebellar contribution to both procedural and declarative components of spatial cognition. To do so, we model a functional interplay between the cerebellum and the hippocampal formation during goal-oriented navigation. We reinterpret and complete existing genetic behavioural observations by means of quantitative accounts that cross-link synaptic plasticity mechanisms, single cell and population coding properties, and behavioural responses. In contrast to earlier hypotheses positing only a purely procedural impact of cerebellar adaptation deficits, our results suggest a cerebellar involvement in high-level aspects of behaviour. In particular, we propose that cerebellar learning mechanisms may influence hippocampal place fields, by contributing to the path integration process. Our simulations predict differences in place-cell discharge properties between normal mice and L7-PKCI mutant mice lacking long-term depression at cerebellar parallel fibre-Purkinje cell synapses. On the behavioural level, these results suggest that, by influencing the accuracy of hippocampal spatial codes, cerebellar deficits may impact the exploration-exploitation balance during spatial navigation.

## Introduction

The cerebellum is known to mediate sensorimotor adaptation [1]–[3], fine movement and coordination control [2], [4], [5], and instrumental conditioning [6]–[8]. However, its role in higher-level functions remains partially understood and controversial [9], [10]. Recent anatomical studies demonstrate that cerebellar outputs target non-motor cortical areas, providing the substrate to influence cognitive tasks [11]. Moreover, the class of functions associated to cerebellar activation has become very diverse and includes language, attention, and emotion related processes [10], [12], [13]. Here, we investigate the role of the cerebellum in spatial cognition, which involves parallel information processing, relational memory, and context-dependent action selection [14]–[17]. We set forth a neurocomputational framework to provide a comprehensive interpretation of behavioural findings supporting the cerebellar implication in spatial navigation [18]–[21]. The presented approach cross-links different organisation levels (e.g. from synaptic plasticity to spatial behaviour) to investigate the functional interplay between the cerebellum and the hippocampal formation during goal-oriented navigation tasks.

Spatial cognition requires both declarative and procedural learning in order to elaborate multimodal representations supporting navigation [22]. Declarative learning allows spatiotemporal relations between multiple cues or events to be encoded [23], [24], and it is instrumental to the formation of cognitive maps for navigation [25]. A large body of experimental work has provided evidence for a role of the hippocampal formation in declarative spatial learning [15], [17], [23], [26]–[32]. The involvement of procedural learning in spatial cognition is more complicated and several aspects must be taken into account. First, procedural learning mediates associations between environmental stimuli and responses, e.g. turning left at the centre of a cross maze, or following a visible cue that changes position from trial to trial in the water maze [22], [33]–[35]. This type of procedural learning is tightly linked to reward-related signalling in the brain and is primarily subserved by the basal ganglia [22], [35], [36]. Second, at a lower level, procedural learning mediates the acquisition of an ensemble of sensorimotor procedures necessary to perform navigation and to optimise goal-directed trajectories (locally in space and time) through sensorimotor adaptation [18], [31]. A further aspect relates to the role of the low-level sensorimotor procedures in global behaviour. Indeed, disturbances in sensorimotor adaptation can have profound influence on high-level aspects of behaviour such as environment exploration [18], [31], [37], ability to perform path integration [38]–[40] and, ultimately, the ability to form a representation of space – the core of declarative spatial memory [41]. The focus of the present study is the link between the low-level, or *local*, procedural learning (i.e. its latter two aspects) and its high-level, or *global*, implications in spatial behaviour and declarative memory.

A growing number of studies suggest an important role of the cerebellum in procedural spatial learning [18], [19], [21], [42]–[46]. For instance, Petrosini et al. [18] demonstrated that hemicerebellectomised rats are impaired in learning effective exploratory behaviour when solving open-field navigation tasks – although their swimming performance is not affected compared to their control littermates. Leggio et al. [44] showed that local procedural mnemonic processes subserving fine tuning of navigation trajectories may involve the interaction between the cerebellum and sub-cortical areas. More recently, Burguière et al. [21] reported that L7-PKCI transgenic mice – which lack parallel fibre-Purkinje cell long-term synaptic depression, LTD [47] – are impaired in the acquisition of optimal goal-directed trajectories, which corroborates the hypothesis that cerebellar LTD may mediate a local sensorimotor adaptation process shared by motor and spatial learning functions [21], [44]. It is worth mentioning that a declarative role of the cerebellum in spatial cognition has also been postulated [41]. By using lurcher mutant mice – which exhibit a massive loss of neurons in the cerebellar cortex and the inferior olivary nucleus – Hilber et al. [41] suggested that cerebellar learning may play a crucial role in the retention of spatial information. However, these results could be ascribed to the strong interaction between procedural and declarative learning [37], [42], [45], [46]. To stress the fact that declarative spatial learning requires appropriate procedural capabilities, Mandolesi et al. [37] showed that hemicerebellar rats are unable to represent a new environment because they can not explore it effectively, although they can detect environmental changes as efficiently as control animals.

A large number of theoretical studies have investigated the cerebellar contribution to adaptive motor control and procedural learning [48]–[52]. By contrast, to the best of our knowledge, no neurocomputational study has addressed the role of the cerebellum in spatial cognition, and a comprehensive interpretation of all aforementioned experimental findings on the cerebellar role in spatial learning is still lacking. Among others, the following issues remain open: can a purely local motor adaptation deficit (i.e. only affecting the low-level component of procedural learning) explain all observed impairments in cerebellar subjects? Is the cerebellum also involved in high-level aspects of procedural spatial learning? Does (and if yes to what extent) cerebellar learning contribute to the declarative component of spatial cognition? In this paper we address these questions by interpreting available experimental data within a quantitative theoretical framework. We attempt to shed light on the cerebellar role (either direct or indirect) in the multiple processing stages mediating spatial learning and goal-directed navigation. The rationale is to complete the existing behavioural observations with quantitative accounts testing specific hypotheses on the link between synaptic plasticity mechanisms, cell discharge properties, interstructure coupling, and behavioural responses.

**Figure 1:**
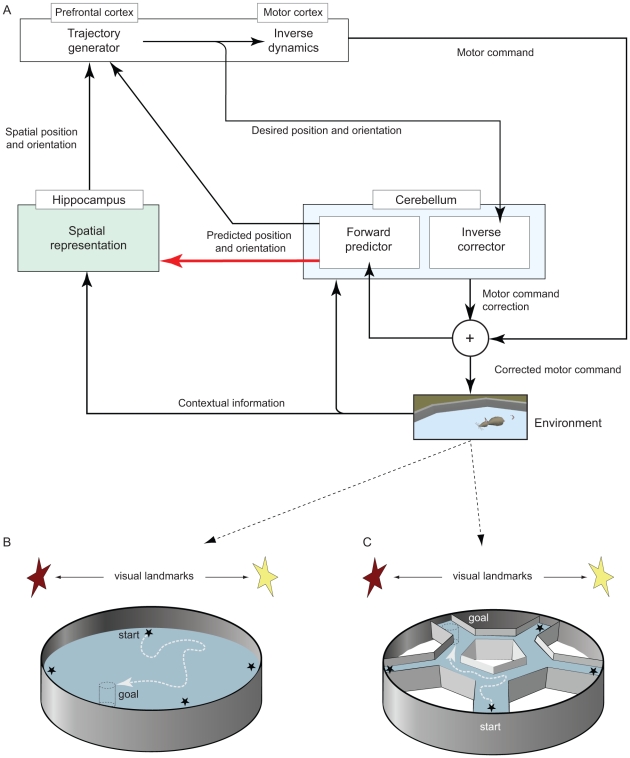
Model architecture and simulated navigation protocols. **A.** Overview of the connectionist model implementing a functional coupling between cerebellar and hippocampal networks. Note that arrows indicate functional projections, which do not necessarily correspond to direct anatomical pathways. **B.** The simulated Morris watermaze [26] consists of a circular maze of 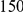 cm in diameter. **C.** The simulated Starmaze [54] is also a circular maze (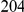 cm in diameter) but it contains alleys (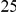 cm in width) forming a central pentagonal ring with radiating arms from each vertex. Both tasks require simulated animals to reach an escape platform (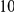 cm in diameter) hidden below the surface of opaque water at a fixed location (black dashed cylinder). At each trial, animals start from one location that is randomly drawn from four possible starting locations (black stars). In both tasks animals can use available visual landmarks (coloured stars) as well as self-motion cues to learn allocentric spatial representations.

To study these questions, we construct a large-scale neural network (Fig. 1A) accounting for the functional coupling between the cerebellum and the hippocampal formation. The modelled architecture also includes a putative cortical module for trajectory planning and inverse dynamics computation. The presented work focuses on the behavioural genetic findings reported by Burguière et al. [21], which suggest that LTD at the parallel fibre-Purkinje cell (PF–PC) synapses is relevant to the adaptive tuning of navigation trajectories. We model the main information processing stages of the cerebellar microcomplex and we emulate the lack of LTD at PF–PC synapses of L7-PKCI transgenic mice [47]. We simulate the experimental protocols employed by Burguière et al. [21] to compare the learning performances of L7-PKCI mutants with those of control animals in two spatial navigation tasks: the Morris water maze [53] (see simulated setup in Fig. 1B) and the Starmaze task [54] (Fig. 1C). In both setups, mice have to swim from random departure locations to a platform hidden below the surface of opaque water. Both tasks require declarative learning to build a spatial representation of the environment. Yet, in contrast to the Morris water maze task, the Starmaze alleys guide movements, which eventually reduces the low-level procedural demand of the task. Thus, the use of these two paradigms allows the relative importance of the declarative and procedural components of navigation to be assessed [21].

Our simulation results suggest that by contributing to the integration of idiothetic (self-motion) cues – i.e. path integration or dead reckoning [16], [55]–[58], cerebellar learning can influence hippocampal spatial representations. We predict changes on the level of single hippocampal cell properties in control *vs*. mutant animals. These cerebellum-dependent changes in spatial coding may in turn lead to behavioural differences between control and L7-PKCI mice, expressed in differences in circling behaviour and exploration-exploitation balance during goal-oriented tasks and free exploratory behaviour.

## Materials and Methods

### 2.1 Integrated model of procedural and declarative spatial learning

Figure 1A shows an overview of the connectionist architecture developed for this study. The core of the model is the functional coupling between the cerebellar and hippocampal networks, which allows the interplay between procedural and declarative components of spatial learning to be investigated. In the following, we first outline the cerebellar microcomplex and hippocampal network models, focusing on the connectivity layout and input-output functional relations. More comprehensive accounts – including neuronal model equations and parameter settings – can be found in the Supplementary Methods S1. Second, we describe the high-level controller and the spatial behaviour policy. Third, we present the simulated experimental setups and protocols. Finally, we describe the statistical analyses assessing both spatial behaviour and neural coding properties.

#### 2.1.1 Cerebellar microcomplex model

In agreement with Marr-Albus-Ito theory [59]–[61], we assume that the cerebellum can acquire internal models of complex sensorimotor interactions [62], [63] and store them in multiple and coupled microcomplexes – the computational units of the cerebellum [64].

Our cerebellar model is composed of six microcomplexes, such that each one constructs an internal model of a particular sensorimotor interaction by adapting its input-output dynamics through online learning [63], [65]–[71]. In particular, two of the six microcomplexes (referred to as *forward predictors* in what follows) construct forward models that predict changes in egocentric orientation and position, respectively, of the simulated mouse, given motor commands that the mouse is about to implement. The other four microcomplexes (*inverse correctors*) learn to map desired future positions into corrective velocity commands compensating for noisy dynamics – and, consequently, for otherwise inaccurate movement execution (e.g. local drifts in swimming trajectories). Both types of internal models mediate low-level procedural spatial learning by encoding the causal relationships determining sensorimotor couplings during navigation.

**Figure 2:**
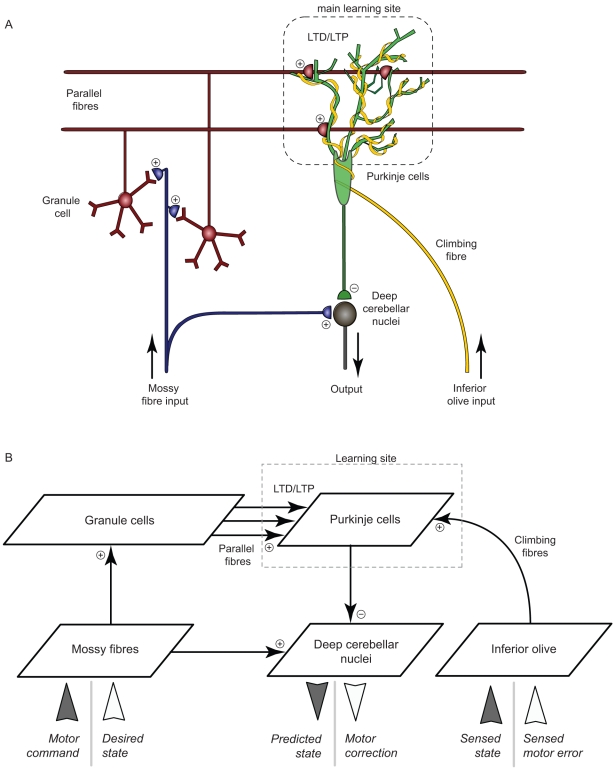
Cerebellar microcomplex model. **A.** A simplified scheme of the cerebellar microcomplex (adapted from [114]). Information enters the cerebellum via two neural pathways: the mossy fibres convey multimodal sensorimotor signals, whereas climbing fibres are assumed to convey error-related information. Granule cells process and transmit sensorimotor inputs to Purkinje cells. Error-related signals also converge onto Purkinje cell synapses, which undergo long-term modifications (i.e. long-term potentiation, LTP, and depression, LTD). **B.** Model cerebellar microcomplex circuit. Each box indicates a population of spiking neurons. The same cerebellar circuit implements both forward (dark gray inputs) and inverse (white inputs) internal models.

Each of the six simulated cerebellar microcomplexes has the same neural architecture, which is inspired by the anatomical properties of the biological cerebellar network (Fig. 2A). We model the basic elements of the cerebellar microcircuit by means of a network of populations of spiking neurons (Fig. 2B). Input signals enter the network via the mossy fibres (MFs), which are connected by excitatory synapses to the granule cells (GCs) and to the deep cerebellar nuclei (DCN). Purkinje cells (PCs) receive excitatory inputs from both GCs (via the parallel fibres, PFs) and inferior olive (IO) neurons (via the climbing fibres, CFs). The PCs inhibit the DCN units, which are the output neurons of the microcircuit. A comprehensive account of the employed coding scheme is detailed in the Supplementary Methods S1, and it is illustrated in Figs. S1 and S2, for the inverse and forward model, respectively.

The basic learning principle in this network is the following. MFs excite DCN neurons via all-to-all constant connections. Without inhibition from PCs, the output of such a defective microcircuit is constant and does not depend on the input. In order for the output to be meaningful, the strength of inhibitory output of the PCs should depend on the input conveyed by MFs via GCs and PFs. The GC layer provides a sparse representation of the MF inputs – the number of GC neurons is 100 times larger than that of MFs and the MF–GC connection probability is only 0.04 (i.e. each MF innervates 400 GCs and each GC receives 4 MF afferents, on average, in agreement with anatomical data [72]–[74]). A sparse representation serves to optimise encoding capacity and information transmission from MFs to PCs [75]. The synapses between PFs (i.e. GCs' axons) and PCs are the only plastic synapses in the model microcircuit, and they learn to translate the (sparsely represented) input into PC output (that inhibits DCN). Bidirectional long-term plasticity (i.e. potentiation, LTP, and depression, LTD) modifies the efficacy of PF–PC synapses and shapes the input-output dynamics of the microcomplex. We implement LTP as a non-associative mechanism [76], such that every incoming PF spike triggers a synaptic efficacy increase. The modelled LTD is a supervised associative mechanism with the teaching signal conveyed by the CFs (output fibres of the IO neurons). This is in accordance with experimental data showing that conjunctive inputs to a PC from PFs and CFs tend to depress the PF–PC projections [61], [77], [78]. To model L7-PKCI transgenic mice, in which PF–PC LTD is not functional [47], we switch off the associative LTD in the modelled PF–PC synapses.

#### 2.1.2 Hippocampal model

The spatial representation module consists of a hippocampal network adapted from our previous works [79]–[81]. The model integrates multimodal spatial information to establish and maintain hippocampal place field representations. The discharge properties of model hippocampal neurons are consistent with those of their biological counterpart [81]. Unsupervised Hebbian learning shapes the dynamics of the hippocampal network producing spatially-tuned neural activity [23]. After training, hippocampal population coding supports place recognition and long-term spatial memory [79], [81].

**Figure 3:**
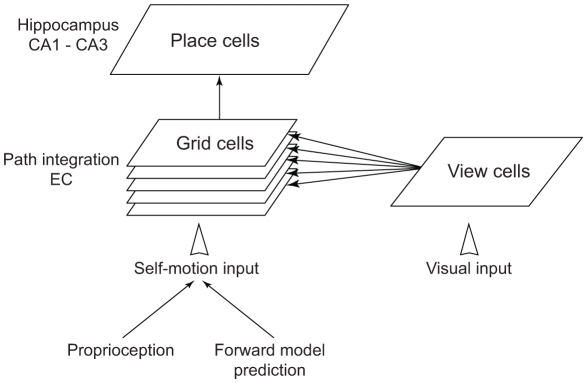
Hippocampal model. Visual input is processed by a set of filters (not shown) that project to hypothetical view cells. Grid cells in the entorhinal cortex (EC) receive self-motion input and visual input, preprocessed by the population of view cells. The grid cells connect to place cells in the hippocampal areas CA1–CA3. Adapted from [81].

Figure 3 depicts a simplified view of the hippocampal model [79]–[81]. The model integrates idiothetic (self-motion) and allothetic (visual landmark related) information to establish and maintain hippocampal place fields. The idiothetic input to model CA1 place cells is provided by feed-forward connections from a population of grid cells in a simulated Layer II of the dorsomedial entorhinal cortex [82], [83]. Model grid cells discharge as a function of integrated self-motion cues over time (i.e. path integration), where self-motion cues represent the velocity vector corresponding to the last movement. The allothetic input is conveyed by panoramic visual snapshots of the environment, processed by a large set of orientation-sensitive filters [80]. This visual information is encoded in a population of vision-based place cells (VC) [81]. As exploration of a novel environment proceeds, unsupervised Hebbian learning allows the hippocampal place field representation to be built incrementally. Since path integration is vulnerable to cumulative error [57], maintaining allothetic and idiothetic representations consistent over time requires to bound dead-reckoning errors by occasionally resetting the path integrator [16], [79]. We assume that the uncertainty of the location estimate provided by the path integrator grows linearly with time. In order to decrease the uncertainty, the simulated mouse uses the learnt allothetic spatial representation – encoded by the VC population activity – to localise itself and calibrate the path integrator, whenever it finds a previously visited location (see [79]–[81], for a full account of the hippocampal model, its implementation details and a validation of the model in a different set of tasks).

#### 2.1.3 Modelling the cerebellar-hippocampal interaction

In this paper, we test the hypothesis that cerebellar procedural learning may influence path integration – and consequently the encoding of idiothetic cues in spatial memories. To do so, we employ the output of the cerebellar forward models (which predicts movement-related sensory feedback) to feed the idiothetic component of our hippocampal place code. In other words, we connect the output of the forward predictors to the path integrator (Fig. 3). Hence, the simulated forward models provide the hippocampal formation – and in particular the medial entorhinal cortex – with self-motion related predictions suitable to refine the estimate of linear and angular displacements over time. We assume that the angular and linear displacements predicted by the forward models for each motor command are combined with sensory self-motion feedbacks (e.g. proprioceptive) to drive the entorhinal grid cell population of the model. Under this scenario, an impaired cerebellar processing would affect path integration and, consequently, the elaboration of hippocampal spatial representations. As a consequence, simulated control and L7-PKCI mice do not share equivalent spatial representation abilities mediated by the hippocampal formation.

#### 2.1.4 Spatial behaviour

The model also includes a high-level module mediating cortical-like action selection and primary motor control (Fig. 1A, see [84] for the role of the prefrontal cortex in action selection and motor control). This module receives as inputs both an estimate of the current state from the hippocampal network and a prediction of the next state from the cerebellar forward models. It plans goal-directed trajectories and it locally maps desired positions onto motor commands through inverse dynamics computation. This module is purely algorithmic, since modelling goal-oriented navigation planning was out of the scope of this paper (see [85] for a recent model of action planning in a prefrontal cortical network model).

Simulated mice select actions (i.e. egocentric motion directions in the range 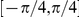) based on a probabilistic policy. At each simulation step 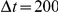 ms, a probability 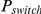 makes the animal select one of the following behavioural responses:

- It can adopt a circling behaviour with probability 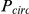 (in the Starmaze environment, where no circling can occur, 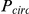 is always zero). As observed by Fonio et al. [86], this peripheral round-trip behaviour is predominant in mice which have not fully explored the near-wall portions of an environment. To account for this observation, we implement 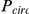 as a function of the amount and the quality of the spatial knowledge of a 10-cm peripheral annulus of the maze:
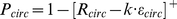
(1)where 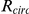 denotes the fraction of the 10-cm peripheral annulus properly encoded by the place cell population activity, and 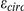 is the mean place code accuracy over the peripheral annulus (see Supplementary Methods S2, Eqs. S7–S8, for the definition of measures 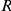 and 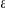); 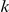 is a normalisation factor and 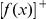 is the positive part operator – i.e. 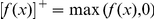. According to Eq. 1, if the near-wall area is well explored (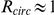) and the spatial localisation error is low (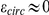), then no circling occurs.
- With probability 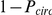 it chooses to either explore the environment or exploit the acquired knowledge. More specifically:
  - It can select an exploratory (random) motion direction with constant probability 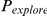.
  - It can exploit the acquired spatial knowledge to perform goal-directed navigation with a probability 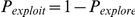. During exploitation, a trajectory planner estimates the direction to the hidden platform (goal) at each time step, based on the hippocampal place code.
- Otherwise, with probability 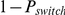, the simulated animal moves in the same direction as in the previous time step. The default values of these parameters are: 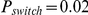; 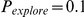. These values allow the stochastic action selection policy to approximate the exploratory behaviour of control mice – in both the MWM and the Starmaze tasks described below.

The overall spatial behaviour model is based on the data from Fonio et al. [86], showing that mice's exploratory patterns consist of reiterated home-centred round-trips of increasing amplitude and “degrees of freedom”. First, mice explore a restricted area around their home base (dimension 0); second, they start moving along the wall of the environment (dimension 1; peripheral round-trip or circling); third, they begin making incursions to the centre of the environment (dimension 2) to fully explore it. Importantly, the exhaustion of a given spatial dimension is a necessary condition for the emergence of the next dimension in the sequence [86].

### 2.2 Spatial navigation tasks and protocols

#### 2.2.1 Morris Water Maze and Starmaze tasks

We test the model against experimental findings in two spatial learning paradigms: the Morris Water Maze (MWM) [53] and the Starmaze task [54] (Figs. 1B, C). We reproduce the experimental protocols used by Burguière et al. [21] to assess to what extent simulated L7-PKCI transgenic mice are impaired, compared to controls, in solving the MWM and the Starmaze. Two groups of simulated mice (n = 15 controls and n = 15 mutants) undertake 4 training trials per day, over 10 days for the MWM and 13 days for the Starmaze. At the beginning of each trial the simulated animal is placed at a departure point randomly drawn from a set of four possible locations (Figs. 1B, C). Each trial ends either when the animal has reached the hidden platform or after a 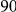 s timeout – i.e. if the animal fails to locate and swim to the platform. Distinct simulated mice in the same group differ in two ways. First, each animal is endowed with a new instance of the cerebellar network, which is initialised according to probabilistic parameters – as described in Supplemenary Methods S1. Second, the spatial policy governing high-level action selection is probabilistic – as described in Sec. 2.1.4. For instance, since explorative *vs*. exploitative responses depend on stochastic variables, it is unlikely that two distinct animals have equivalent navigation trajectories. These differences in spatial behaviour impact, in turn, the information content of the hippocampal place code, which is built incrementally and depends on the exploration-exploitation balance of a given simulated animal.

We simulate the MWM and the Starmaze experimental protocols in the Webots platform [87]. The latter provides a realistic environment where simulated animals can process visual, proximity (whisker-like), and self-motion (proprioceptive-like) signals. Simulated mice move at a speed within the range of 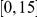 cm/s. Sensory feedback (e.g. visual, tactile and proprioceptive information) occurs every 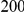 ms in order to process internal state variables and select actions. Prior to the action execution, an inverse dynamics module (Fig. 1A) translates actions into low-level motor commands. Stochastic noise affects the execution of each action, emulating unpredictable sensorimotor perturbations and/or drifts from desired swim trajectories.

### 2.3 Statistical analyses

#### 2.3.1 Behavioural analysis

We compare the goal-oriented behaviour of L7-PKCI and control mice by assessing the same set of parameters measured by Burguière et al. [21]: *(i)* the mean escape latency (s), i.e. the average time spent to reach the platform; *(ii)* the mean heading (deg), computed as the average angular deviation between the ideal and actual trajectory to the goal; *(iii)* the mean circling time (s), i.e. the average time spent in a 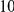-cm peripheral annulus of the maze [44]; *(iv)* the ratio between the time spent in the target quadrant and the trial duration; *(v)* the mean distance-to-goal (cm), i.e. the average Euclidean distance between the animal and the platform; *(vi)* the mean distance swum by the animal (cm); *(vii)* the search score, characterising the shape of a goal-oriented trajectory [43]; *(viii)* the mean number of visited alleys (for the Starmaze only); *(ix)* the mean speed of the animal (cm/s). We average each parameter over all trials performed in one day by all subjects of a same group. An ANOVA analysis quantifies the statistical significance of the results (with 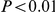 considered as significant).

#### 2.3.2 Analysis of unitary and population neural activities

We characterise the activity patterns of unitary and multiunit discharges in terms of spatial encoding properties and time course of the spatial learning process. We quantify: *(i)* the spatial selectivity properties of single cells by measuring the coherence [88], mean size, and number of peaks of the receptive fields; *(ii)* the density – and other correlated measures such as sparseness and redundancy – of the population place code; *(iii)* the reliability of neural representations (at the level of both single cell and population codes) in terms of spatial information content – i.e. how much can be inferred about the animal's position by observing the neural responses only; *(iv)* the time course of the accuracy of the population vector estimate for the animal's position [89], [90]; *(v)* the time course of the mean percentage of locations appropriately encoded by the spatial representation – i.e. the explored locations where the accuracy of the population vector estimate is above a fixed threshold. An ANOVA analysis measures the statistical significance of the results (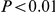 is considered as significant). See Supplementary Methods S2, for details on the statistical measures and parameters employed for data analysis.

We assess the overall accuracy of the spatial representation by means of two complementary measures, namely 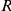 and 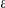, which quantify the amount of information encoded in the hippocampus and the quality of this information, respectively. The measure 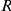 estimates the percentage of the environment covered by the place field population and is calculated as the fraction of positions where the animal can self-localise (and then recalibrate its path integrator) with good accuracy (the accuracy threshold is set to 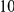 cm). The measure 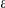 quantifies the accuracy of the place code as the mean self-localisation error – i.e. the discrepancy between the actual position of the animal and the position estimated by population vector decoding of hippocampal activity [89], [90]. See Supplementary Methods S2, for the definition of measures 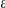 and 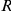.

## Results

During training in the MWM, adaptation in cerebellar forward models resulted in correct predictions of sensory outcomes of given motor commands (see Supplementary Results S1, Figs. S3 A–E), whereas learning in simulated inverse correctors improved the accuracy of motor command execution significantly (Supplementary Results S1, Figs. S3 F–J). Sensorimotor adaptation in both models relied on the associative LTD in the PF–PC synapses of the cerebellar network (see Fig. 2). Consequently, the performance of simulated L7-PKCI mutants, in which this LTD is absent, was inferior to that of simulated controls, and this deficit was evident from the first day of training (Figs. S3 C, H).

In the context of the full spatial learning model (Fig. 1), we expected L7-PKCIs' cerebellar adaptation deficit to have two primary consequences. First, it could influence low-level (or local) motor behaviour. Indeed, an impairment in the inverse corrector component would cause deviations in the performed motor commands relative to the desired ones, leading to inaccurate implementation of goal-directed trajectories in mutants, relative to controls. Second, it could disrupt the normal functioning of the cerebellar-hippocampal interaction, potentially influencing higher-level (or global) spatial learning and behavioural responses – i.e. beyond purely cerebellar sensorimotor adaptation functions. To dissociate the roles of cerebellar adaptation deficits in local *vs*. global spatial behaviour, we tested the full cerebellar-hippocampal model in the experimental paradigm proposed by Burguière et al. [21], which allows the relative contributions of procedural and declarative components of spatial navigation to be assessed.

### 3.1 Cerebellar role in declarative spatial learning

#### 3.1.1 Cerebellar adaptation deficits impair goal-directed navigation

**Figure 4:**
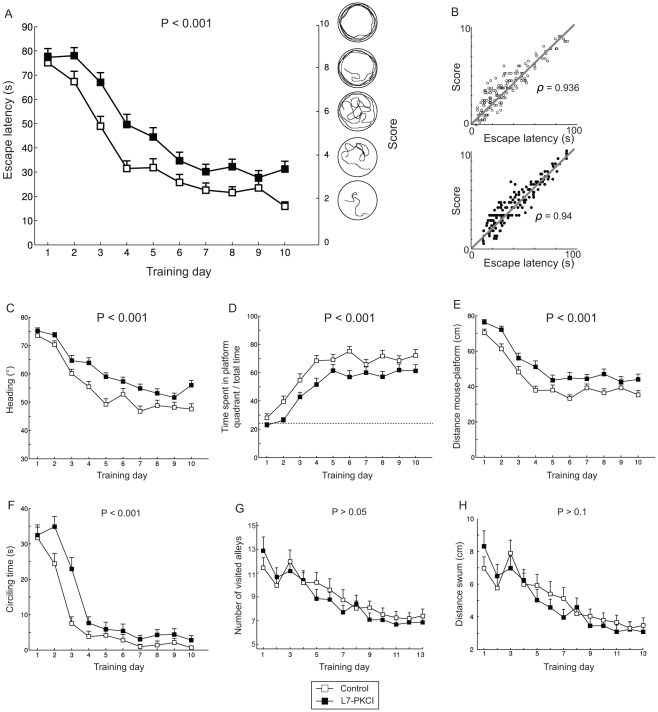
The hypothesis of a cerebellar influence on path integration, and hence on hippocampal place coding, accounts for all L7-PKCIs' spatial navigation impairments observed experimentally. **Results in the MWM:** **A.** Mean escape latency over training (left y-axis) and score (right y-axis) of simulated controls and mutants. **B.** Correlation between searching score and escape latency for control (top) and mutant (bottom) simulated mice. **C.** Mean angular deviation between ideal and actual trajectory to the goal. **D.** Ratio between the time spent in the platform quadrant and the total duration of a trial. **E.** Mean distance of the simulated mouse to the platform. **F.** Mean circling time. **Results in the Starmaze:** **G.** Mean number of visited alleys. **H.** Mean distance swum in the Starmaze.

We tested simulated controls (i.e. with intact cerebellar learning) and L7-PKCIs (i.e. with disabled cerebellar LTD) in the MWM task. Both the mean escape latency and the search score of simulated mutants were significantly impaired over training compared to controls (Fig. 4A; escape latency: ANOVA, 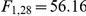, 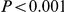; search score: ANOVA, 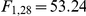, 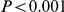). These two behavioural measures were highly correlated for both groups of simulated animals (Fig. 4B; controls: Pearson's product-moment coefficient 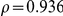, 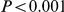; mutants: 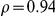, 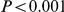). This navigation impairment was not due to a deficit in swimming speed (not shown, ANOVA, 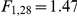, 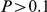). Navigation trajectories of simulated L7-PKCIs were also significantly less efficient than controls in terms of heading-to-goal – i.e. deviation between actual and direct trajectory to the platform (Fig. 4C, ANOVA, 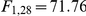, 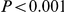). The intergroup differences of searching behaviour were also corroborated by the ratio between the time spent within the platform quadrant and the trial duration, showing a significant impairment of simulated mutants (Fig. 4D, ANOVA, 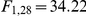, 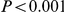). Similarly, L7-PKCIs' spatial behaviour led to significantly longer mean distance-to-goal over training than controls (Fig. 4E, ANOVA, 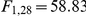, 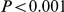). The circling time of simulated L7-PKCI mice was significantly larger, compared to controls, over the entire training phase (Fig. 4F, ANOVA, 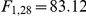, 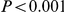). Since the action selection policy (Sec. 2.1.4) was exactly the same in controls and mutants, the observed intergroup difference in circling time indicates that mutants needed significantly more time than controls to acquire an accurate spatial representation of the peripheral areas of the environment [86].

**Figure 5:**
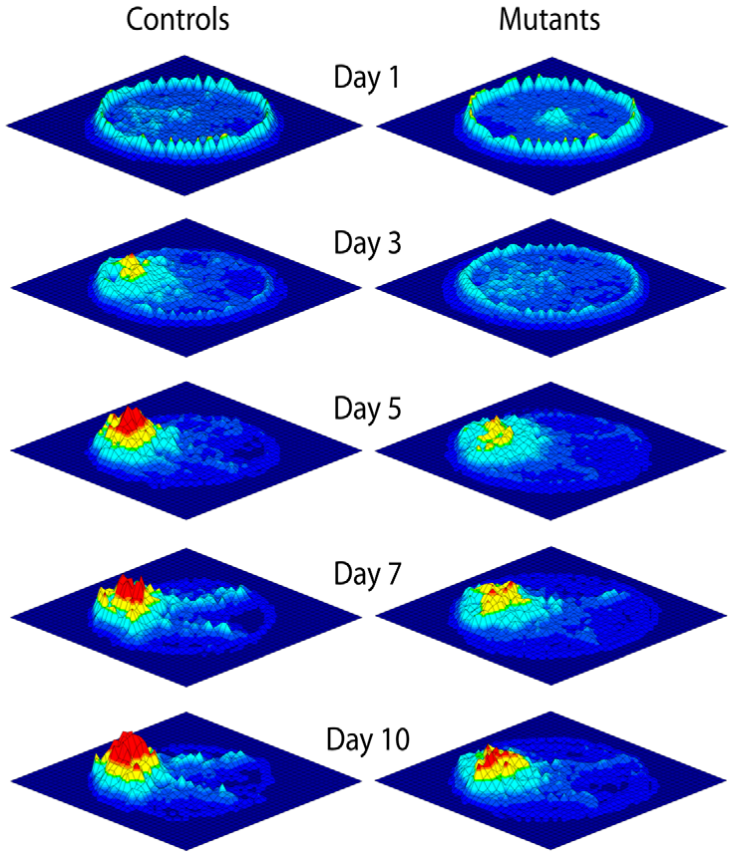
The hypothesis of a cerebellar influence on path integration, and hence on hippocampal place coding, accounts for all L7-PKCIs' spatial navigation impairments observed experimentally. Occupancy maps in the MWM. Three-dimensional diagrams of the mean time spent by control and mutant mice at each location of the maze at different training phases (days, 1, 3, 5, 7 and 10). A grid of resolution 31 × 31 (each grid cell is 5 × 5 cm) samples spatial locations. The value associated to each grid cell is the normalised time spent in the cell region with respect to the duration of each trial, averaged over all day trials and over all animals of a group.

Figure 5 visualises behavioural differences between simulated controls and mutants qualitatively, by showing the occupancy plots for simulated controls and mutants. Both simulated groups improved their spatial behaviour through training and succeeded in localising and navigating to the platform from any starting location of the maze. Yet, consistently to quantitative results of Figure 4, mutants exhibited a longer circling time and a wider spread in searching behaviour than controls.

Overall, these simulation results fully account for the navigation impairments of L7-PKCI mice in the MWM observed experimentally [21]. Also, the mean intergroup differences are comparable in simulation and experiments for all measured behavioural parameters (escape latency: ANOVA, 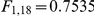, 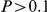; heading: ANOVA, 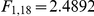, 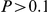; ratio between time spent in the platform quadrant and trial duration: ANOVA, 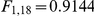, 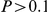; distance to the platform: ANOVA, 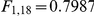, 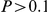; and circling time: ANOVA, 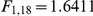, 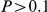).

In contrast to the MWM task and in agreement with data from Burguière et al. [21], the same control and mutant groups did not exhibit any significant difference in the simulated Starmaze task (Figs. 4G,

H). We did not observe any statistically significant intergroup difference in terms of mean number of visited alleys (Fig. 4G, ANOVA, 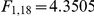, 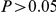), mean distance swum to reach the platform (Fig. 4H, ANOVA, 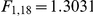, 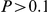), and mean escape latency (not shown, ANOVA, 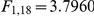, 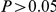).

The above simulation results suggested that our cerebellar-hippocampal model could reproduce the navigation behaviour of control and L7-PKCI mice in both MWM and Starmaze tasks. However, the question remained of whether the navigation impairment of mutants in the MWM task was due to a purely local adaptation deficit, as suggested by Burguière et al. [21], or also to a more global deficit induced by an ineffective cerebellar-hippocampal interaction. To answer this question, we blocked the functional input from the cerebellum to the path integration network (Fig. 1), in order to isolate the local procedural and the global declarative components of the spatial learning model. Our simulation results indicated that a purely local sensorimotor adaptation deficit could not account for all L7-PKCIs' navigation impairments observed experimentally (see Supplementary Results S2, Figs. S4, S5). In particular, the goal-searching behaviour of simulated mutants was not significantly impaired by the local procedural deficit only (Figs. S4 C, D). Thus, our results suggested that a functional connection between the cerebellum and the hippocampal formation is necessary to explain the spatial behaviour differences between controls and mutants, corroborating the hypothesis of a cerebellar involvement in declarative spatial memory.

#### 3.1.2 Cerebellar adaptation deficits reduce the accuracy of hippocampal place representations

**Figure 6:**
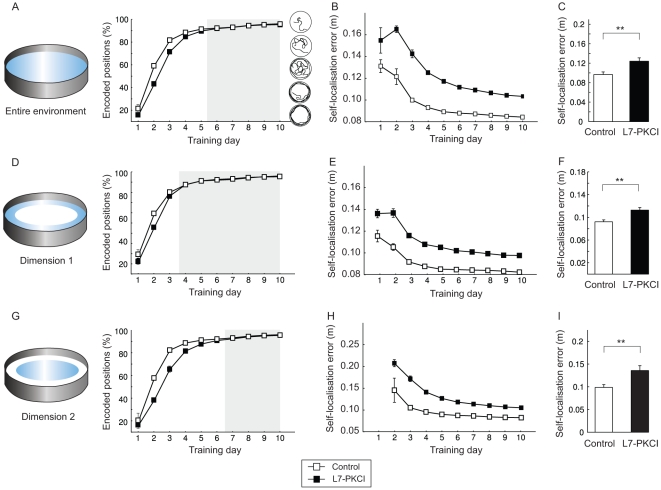
Cerebellar adaptation deficits reduce the rate of acquisition and the accuracy of hippocampal place field representations in the MWM. **A–C.** Time course and accuracy of the hippocampal spatial map (measured in the whole environment) in simulated controls and mutants. The bar diagram in C shows the grand mean of the spatial code accuracy averaged over the entire training. **D–I.** Time course and accuracy of spatial map in the peripheral area (D–F) and the central region (G–I) of the MWM.

We next studied in more detail what properties of the spatial memory function were different in simulated control and mutant mice. We compared the time course of hippocampal spatial information coding in simulated controls and mutants solving the MWM (Fig. 6). Both the rate and the accuracy of hippocampal place coding were impaired in L7-PKCIs, relative to controls, with significant time course differences during days 1–5 of training (Fig. 6A, ANOVA, 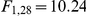, 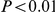). Although both simulated groups improved the accuracy of their spatial code over time (Fig. 6B), simulated mutants exhibited significantly larger self-localisation errors than controls through the entire training (ANOVA, 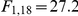, 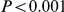). Consistently, this result was confirmed by averaging over all training sessions (Fig. 6C; ANOVA, 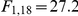, 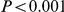). Thus, the spatial code was less accurate in simulated mutants than in controls solving the MWM.

#### 3.1.3 Population place coding is suboptimal in L7-PKCI mice compared to controls

**Figure 7:**
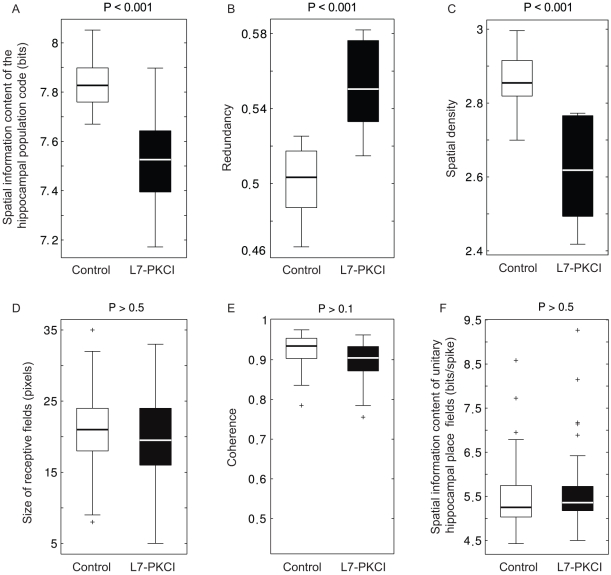
Population place coding is suboptimal in simulated L7-PKCI mice compared to controls. **A.** Information content of the spatial code encoded by the population of hippocampal place cells in controls and mutants. **B.** Redundancy of the hippocampal spatial code in both simulated groups. **C.** Spatial density of hippocampal place fields in both groups. **Unitary hippocampal place fields in simulated L7-PKCI mice are not impaired, relative to controls, in terms of size, spatial coherence and information content. D.** Mean size of place fields in simulated controls and mutants. **E.** Mean spatial coherence (local smoothness) of place fields in both groups. **F.** Mean spatial information content of unitary place fields in both groups.

What were the neural properties on the population level responsible for this difference in accuracy? We further assessed the characteristics of hippocampal population codes in both simulated groups (Figs. 7A–C). The mean spatial information content of controls' place field representation was significantly larger than in mutants (Fig. 7A; ANOVA 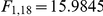, 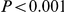). The redundancy of the spatial information content of the two neural population codes tended, on average, to be larger in mutants than in controls (Fig. 7B; ANOVA 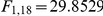, 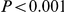). The intergroup difference of mean spatial density of receptive fields confirmed this observation (Fig. 7C; ANOVA 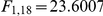, 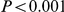). These results pointed towards suboptimal place field representations in mutants – i.e. encoding less spatial information despite a more redundant (dense) place code, compared to controls. But where did this lack of optimality at the level of mutants' population code come from?

#### 3.1.4 Effects of cerebellar adaptation deficits on the properties of unitary hippocampal place fields

To address the above question we compared the spatial tuning properties of single hippocampal place cells in both simulated groups (again when solving the MWM). We observed that the size of hippocampal receptive fields was comparable, on average, in controls and mutants (Fig. 7D; ANOVA 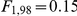, 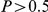). Similarly, the spatial coherence of mutants' place fields was not impaired compared to controls (Fig. 7E; ANOVA 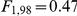, 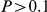). In addition, there was no significant intergroup difference with respect to the amount of spatial information encoded by single hippocampal units (Fig. 7F; ANOVA 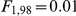, 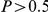). Finally, place cells in mutants and controls were also statistically comparable in terms of mean firing rate (not shown, ANOVA 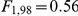, 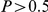). Thus, these standard measures of spatial tuning and accuracy of unitary hippocampal responses failed to explain the subtle but significant difference observed at the level of population spatial coding in controls and L7-PKCIs.

**Figure 8:**
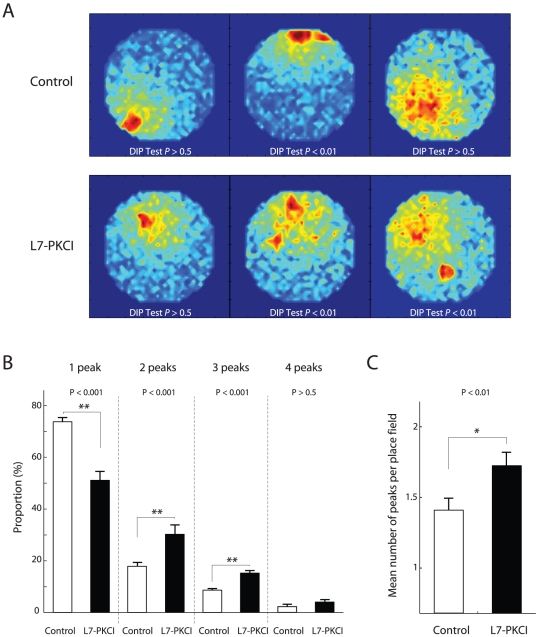
Multipeak place fields occur with higher probability in simulated L7-PKCI than in control mice. **A.** Samples of receptive fields of simulated place cells from control (top) and mutant (bottom) simulated animals. Each plot shows the mean discharge of the recorded neuron as a function of the animal position in the MWM (red and blue denote peak and baseline firing rates, respectively). The unimodality property of firing distributions is statistically assessed by a Hartigan DIP test (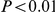 indicates a multipeak receptive field). **B.** Percentage of unimodal and multimodal place fields in simulated controls and mutants. **C.** Mean number of peaks per hippocampal place field in both groups of simulated animals.

We then investigated the properties of model hippocampal single units by quantifying the unimodal *vs*. multimodal characteristics of their spatially tuned discharges. Figure 8A shows some samples of hippocampal place fields “recorded” from simulated controls (top) and mutants (bottom) in the MWM. For each cell, we report the statistical significance of the Hartigan DIP unimodality test [91] used to classify the spatial firing distributions of single hippocampal units (DIP test 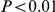 indicates a multi-peak receptive field).

We quantified the ratio between unimodal and multimodal place fields for both simulated groups (Fig. 8B, first column). Simulation results suggested that both groups had a large fraction of single-peak place cells – controls: 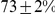, mutants: 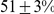. However, they also indicated that, on average, the proportion of single-peak hippocampal receptive fields was larger in controls than in L7-PKCIs (ANOVA 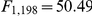, 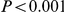). Consistently, mutants had a significant larger ratio of double- and triple-peak place fields than controls (Fig. 8B, second and third column, respectively; ANOVA , 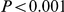 and 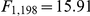, 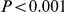, respectively). By contrast, both groups had a negligible percentage of four-peak place fields (Fig. 8B, fourth column). Consistent with previous results, L7-PKCI had, on average, a significantly larger number of peaks per hippocampal place field than controls (Fig. 8C; ANOVA 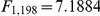, 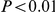). Therefore, the model predicts that this subtle spatial selectivity impairment on the level of unitary hippocampal cells could be responsible for suboptimal spatial population coding in L7-PKCIs, relative to controls, in the MWM. The latter, in turn, could be responsible for the impaired goal-searching behaviour of simulated mutants in the MWM (Figs. 4 D, E).

### 3.2 Cerebellar influence on exploration-exploitation control

Our results suggested that the cerebellar (functional) input to the hippocampal network could contribute to declarative spatial learning by improving the accuracy of path integration and, indirectly, of place field representations. The quality of the spatial code could, in turn, influence the exploration-exploitation balance. Indeed, spatial goal-directed actions rely on accurate place mapping – because solving complex navigation tasks (e.g. the hidden-platform version of the MWM) requires generating goal-directed trajectories based on the knowledge of both the current position of the animal and the target location. Therefore, differences between mutants and controls in the accuracy of the spatial code may result in differences in the initiation of goal-directed actions, i.e. the switch from exploration to exploitation. By testing this hypothesis in simulation, we found that the MWM data by Burguière et al. [21] could indeed reflect an unbalanced exploration-exploitation trade-off in L7-PKCI mice, compared to controls (see Supplementary Results S3, Fig. S6). Our results suggested a significant bias towards explorative behaviour in mutants, in order to compensate for inaccurate spatial learning. On the basis of the above results, we propose the following hypothesis on the role of cerebellar learning in declarative spatial memory. First, cerebellar adaptation mechanisms are likely to influence spatial learning by contributing to the accuracy of path integration, and hence to the quality of hippocampal spatial representations. Second, on the behavioural level, this contribution induces an increased exploration time in mutants, and hence delays the switch from exploration to exploitation in goal-oriented tasks.

**Figure 9:**
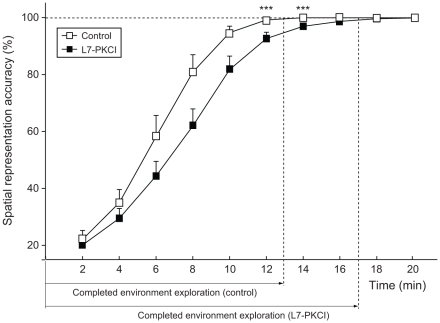
Cerebellar adaptation deficits impact the exploratory behaviour of simulated L7-PKCI mice. Time course of the spatial representation accuracy as a function of exploration time for both simulated controls and mutants undertaking a free-exploration task.

In the context of the free exploration paradigm proposed by Fonio et al. [86], this assumption leads to the following behavioural prediction: mutant mice would need more time, compared to controls, to exhaust the exploration of a given “spatial dimension” (e.g. the near-wall area). To illustrate this prediction in our model, we assessed the free exploratory behaviour of simulated controls and mutants solving a latent spatial learning task in a circular track (10 cm width). We let n = 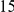 controls and n = 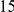 mutants freely explore the circular maze from a fixed starting location (home base). We measured the accuracy of the acquired hippocampal spatial code and compared the time necessary for simulated controls and mutants to exhaustively explore the environment (Fig. 9). In the context of the experiment by Fonio et al. [86], this would correspond to the time required to exhaust dimension 1 (i.e. circling behaviour), a necessary condition for the emergence of incursions into the centre of an open-field environment (i.e. dimension 2). Our simulation results showed that both groups significantly improved their hippocampal space code over time through free exploratory behaviour. However, simulated mutants needed significantly more time than controls to achieve the same place map accuracy (Fig. 9; ANOVA, 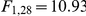, 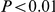). On average, simulated mutants exhausted dimension 1 within 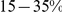 more time than control mice.

## Discussion

This study investigates the role of the cerebellum in spatial navigation. It completes existing experimental findings by quantitatively testing specific hypotheses on the role of the cerebellar-hippocampal interaction, on the link between synaptic plasticity mechanisms, unitary and population discharge properties, and behavioural responses. We focus on behavioural genetic findings by Burguière et al. [21] and we study the functional relevance of plasticity at parallel fibres-Purkinje cell (PF–PC) synapses in navigation tasks. On the one hand, our results corroborate the hypothesis that cerebellar adaptation significantly contributes to the fine tuning of goal-directed trajectories [21]. On the other hand, we draw a novel interpretation of these experimental data. Our results counter the hypothesis of a purely local procedural deficit being entirely responsible for all observed spatial learning impairments of L7-PKCI mice – which lack LTD at PF–PC synapses [47]. Rather, our results suggest that the less efficient goal-searching behaviour of mutants (compared to controls) reflects an implication of the cerebellum in higher-level aspects of spatial learning. In particular, we propose that by providing predictive state information to entorhinal grid cells, the cerebellum may play a role in path integration, and hence contribute to the construction of hippocampal spatial representations.

In view of this proposal, an impaired sensorimotor adaptation at the cerebellar level would delay the formation of coherent spatial maps in the hippocampus. Our simulation results show that L7-PKCI mice are impaired in solving the MWM in terms of both acquisition rate and encoding accuracy of spatial information. Furthermore, our results support the hypothesis that suboptimal spatial representations may lead to suboptimal exploratory behaviour, primarily expressed as a deficit of L7-PKCI mice in inhibiting thigmotaxis and in trading-off exploration and exploitation. Similar to Burguière et al. [21], we find that simulated L7-PKCI mice are not impaired in solving the Starmaze task. Our results suggest that the Starmaze's alleys are likely to reduce the accumulation of path integration errors over time, preventing the declarative component of spatial memory to be impaired in mutants. This conclusion differs from the one drawn by Burguière et al. [21], who interpret the absence of deficit in the Starmaze as a proof that only procedural learning is impacted in L7-PKCI mice. By contrast, we propose that both procedural and declarative spatial learning are affected in L7-PKCI mice. However, in the Starmaze, the impact of cerebellar adaptation deficits on the accuracy of hippocampal place codes is likely not to be significant.

The relative contributions of procedural and declarative components of spatial navigation can be estimated from our results by comparing the performance of simulated mice with (Fig. 4) and without (Fig. S4) the functional connection between the cerebellum and the hippocampal formation. In the MWM, this comparison suggests a stronger contribution of the declarative component to spatial measures related to the knowledge of the goal location (i.e. the fraction of time spent in the platform quadrant and the distance mouse-platform). This is not surprising, since a more accurate spatial map leads to more efficient exploitative actions leading towards the goal.

### The cerebellar role in path integration

Our prediction on the cerebellar role in path integration is in line with earlier proposals [92]–[94]. More recently, Korelusova et al. [95] observed that, in a group of lurcher mice, those mice that in addition suffered from a retinal degeneration were not able to use idiothetic navigation to solve a spatial orientation task. These results suggested that lurcher animals can not integrate self-motion information, and thus reinforce the plausibility of the hypothesis that cerebellar computation can be instrumental in path integration. The hypothesis of a cerebellar role in path integration is also in agreement with recent experimental findings on monkeys demonstrating that the cerebellar circuit is implicated in acquiring and processing information necessary for spatial orientation and self-motion perception [96]–[98]. Shaikh et al. [96] proposed that a role of the cerebellum in spatial orientation could be to transform motion related signals in different reference frames usable to encode body motion. The authors demonstrated that activities of motion-sensitive neurons in the rostral fastigial nucleus have a distributed representation in different reference frames, whereas cells in the vestibular nuclei primarily encode motion in an egocentric reference frame. These results suggest that the cerebellum may transform body coordinates in different reference frames that might be usable to encode body motion. In a more recent study, Yakusheva et al. [97] made a similar observation and showed that cerebellar cortical activity in nodulus and uvula (lobules X and IX of the vermis) reflects the critical computations of transforming head-centred (egocentric) vestibular afferent information into world-centred (allocentric) self-motion and spatial orientation signals. More precisely, the authors showed that Purkinje cells of theses areas encode inertial motion. The Purkinje cells in the nodulus and uvula appear to carry the world-horizontal component of spatially transformed and temporally integrated rotation signals. This transformation appears critical for extracting the inertial linear accelerations during navigation, and thus providing the information to brain areas involved in the retention of spatial memories [97], [98]. Very recently, Rocherfort et al. [99] demonstrated that the hippocampal place code may be impaired when L7-PKCI mice primarily rely on self-motion cues. The authors suggest that cerebellar PKC-dependent mechanisms – such as cerebellar LTD between PF–PC synapses – are involved in the shaping of hippocampal spatial representations. In agreement with our conclusions, the authors postulate that the cerebellum is likely to be involved in the processing of self-motion signals [99].

Anatomical evidence also supports this functional hypothesis. The flocculonodular lobe (the vestibulo-cerebellum) has primary connections with the vestibular nuclei [100], [101], and it also receives visual inputs. The vestibulo-cerebellar tract carries information from the semi-circular canals of the inner ear to the cerebellum via the vestibular nucleus located in the lower pons and medulla. In addition, the reticulo-cerebellar tract conveys signals received by the reticular nuclei in various parts of the brainstem from the cortex, spinal cord, vestibular system and red nucleus. Then, the medial zone of the anterior and posterior lobes (which constitutes the spinocerebellum or paleocerebellum) receives proprioceptive inputs from the dorsal columns of the spinal cord and from the trigeminal nerve [102], as well as from visual and auditory systems [103]. It sends fibres to the deep cerebellar nuclei that, in turn, project to both the cerebral cortex and the brainstem, thus providing modulation of descending motor systems.

The cerebellum is likely to encode the dynamics of body limbs by using this idiothetic information and an efference copy of the motor command. When the cortex sends a motor command to lower motor neurons in the brainstem and spinal cord, a copy of this message reaches the cerebellum through the cortico-pontine-cerebellar tract [103]. There is strong evidence for the cerebellum to predict future states of the limbs by using this efference copy [104]–[106]. As a consequence, it is plausible that the cerebellum provides an estimates of the future state of the whole body (position, orientation, speed) to refine sensory feedback information.

### Cerebellar adaptation influences hippocampal place coding

An important prediction of this work is at the level of neuronal activity of hippocampal place cells. The presented results provide new insights on how dysfunctions at the cerebellar plasticity level could have observable implications on the construction of hippocampal place maps. A possible impact may concern the shaping of place fields, and in particular the multimodal *vs*. unimodal characteristics of spatially selective activity profiles – i.e. the mean number of peaks of hippocampal receptive fields. In our analyses, this discrepancy is one of the signatures of suboptimal population place coding in the simulated L7-PKCI mice. This hypothesis can be tested by performing electrophysiological recordings of pyramidal cells in the hippocampal formation (both CA1–CA3 place cells and entorhinal grid cells) of L7-PKCI mice solving open-field spatial tasks. We expect to have larger unitary place coding differences when idiothetic cues are the main source of information for place learning.

### The role of the cerebellum in exploratory behaviour

Another prediction derived from our results concerns the consequences of suboptimal spatial coding in L7-PKCI mice for exploratory behaviour. In our model, the probability of circling behaviour depends directly on the quality of the hippocampal spatial code. Since simulated L7-PKCI mice have less accurate spatial representations than controls, they express an increased circling behaviour, in agreement with experimental data [18]. Our results suggest a bias in L7-PKCIs' exploration-exploitation balance towards exploration. We predict that in the free exploration paradigm proposed by Fonio et al. [86], L7-PKCI mice should exhibit a delayed switch between “macro degrees of freedom” compared to controls (e.g. from exploration of near-wall areas to incursions into the centre of the environment). According to our model, this observable behavioural differences between mutant and control animals would be due to local procedural deficits and impaired integration of idiothetic movements. Therefore, conducting this experiment in darkness, which increases the importance of idiothetic cues, may result in more significant intergroup behavioural differences.

### Relation to other experimental data

We now discuss and re-interpret available experimental data within our theoretical framework. The first evidence for a cerebellar role in spatial behaviour dates back to Lalonde et al. [42], [92], [93], who assessed the navigation abilities of weaver, staggerer and lurcher mutants in comparison to control mice – weaver mutants present a selective degeneration of cerebellar granule cells; staggerer mice lose cerebellar Purkinje cells, granules cells and inferior olive neurons; and lurcher mutants present a degeneration of the olivo-cerebellar system. All three types of mutants have deficits in the acquisition of maze learning, with different degrees of severity [42], [92], [93]. Although these studies suggested that procedural memory was likely to be primarily affected, they could not dissociate the relative importance of procedural and declarative memories in the observed deficits. Also, the cerebellar ataxia produced by such mutations and the subsequent visuo-motor deficits (e.g. lurcher mutants had difficulties in navigating toward a visible goal [93]) made it difficult to interpret the observed procedural impairments. In the light of our results, we suggest that weaver, staggerer and lurcher mice may have developed local and global procedural impairments. Also, depending on the complexity of the task, mice could have suffered from a delay in the establishment of declarative memories compared to control animals. This would also explain the deficit observed experimentally in maze learning with staggerer mice [92].

A series of studies using hemicerebellectomised (HCbed) rats demonstrated that cerebellar specific lesions impair the development of efficient exploration strategies [19], [44], [107]. These works suggested that the cerebellar network might be involved in the acquisition of all procedural components necessary for optimal spatial behaviour [44], as well as in acquiring new behaviours and in modifying them in relation to contextual information [19]. The authors also examined the influence of cerebellar lesions on spatial exploration in the presence of different spatial distributions of multiple rewards. In all configurations, lesioned animals had impaired exploratory behaviour [107]. Other works have addressed the specific functions of hippocampal and cerebellar networks in learning spatial procedural strategies [108], [109]. The authors investigated the role of NMDA receptors in the exploratory behaviour of rats, and the influence of isolated hippocampal and cerebellar lesions. Leggio et al. [108] reported that in the presence of a lesioned hippocampal formation, the NMDA receptor antagonist influences the acquisition of spatial procedures – it is known that NMDA-dependent LTP in the hippocampus is not essential for spatial procedural learning [110]. Federico et al. [109] showed that the injection of NMDA antagonist mimics the consequences of cerebellar ablation, thus suggesting that the cerebellum – via the activity of NMDA receptors – could be involved in the acquisition of spatial procedures. The conclusions provided by our quantitative study are consistent with all these results and corroborate the hypothesis that both local and global components of spatial cognition are influenced by cerebellar processing – i.e. respectively, a local optimisation of the trajectory, and the efficient learning and use of global procedures for optimally solving a spatial task [44]. However, our study does not address the lack of flexibility in changing behaviours observed in HCbed rats [19], [44].

Petrosini et al. [43] observed that HCbed rats succeeded in navigating towards a platform in pure place learning paradigms – such as finding a hidden platform from sequentially changed starting positions. However, HCbed rats showed significantly lower spatial learning abilities than controls [43]. Subsequent interpretations of these results focused on the cerebellar contribution to procedural spatial learning and neglected possible involvements in building spatial maps [19], [37], [44]. The study presented here postulates a role of the cerebellum in path integration and suggests that an affected idiothetic-based navigation may account for the observed delay in the ability to learn spatial maps – in addition to the local procedural deficit. Our results propose that the declarative-like impairment might only slightly affect rodents in tasks where idiothetic and allothetic information is available, but could be accentuated in tasks where self-motion related signals are the main source of information, or in paradigms where the two types of cues are set in conflict.

In another study using HCbed rats, Mandolesi et al. [37] investigated the relationship between procedural and declarative spatial knowledge. The authors pointed out that no declarative learning was possible without appropriate procedural spatial learning: HCbed rats were not able to represent a new environment because they were not able to explore it appropriately [37]. It is interesting to call attention to a possible reverse interaction between procedural and declarative memories supported by data demonstrating the emergence of a stereotyped exploration behaviour in freely moving animals [86]. In the light of our results, we make the hypothesis that rodents can not appropriately explore the environment, unless they can efficiently represent the dimensions of this environment. In the experiment by Burguière et al. [21], this is illustrated by mutants' longer thigmotaxic behaviour compared to controls. Our results indicate that this difference can be understood in terms of the interaction between declarative and procedural memories, suggesting that the inhibition of a circling behaviour will be favoured by a better knowledge of the near-wall portions of the environment. Therefore, we extend the observation made by Mandolesi et al. [37], and propose that a deficit in procedural components when performing a navigation task should be taken carefully, and would sometimes need to be discussed in terms of a possible influence of the declarative learning on procedural memories.

### Limitations of the model

In our model, both LTD and LTP at PF–PC synapses allow the simulated cerebellar microcomplex to learn sensorimotor associations during spatial navigation. This view is in line with previous proposals on the role of cerebellar LTD in procedural learning and, in particular, in motor control adaptation [111]. Importantly, recent works have re-examined the functional role of cerebellar PF–PC LTD and suggested that this synaptic plasticity mechanism might not be essential to motor learning. Schonewille et al. [112] tested three different types of mutant mice lacking PF–PC LTD in numerous cerebellar-dependent coordination tasks and did not observe any motor learning impairment. Alternatively, the authors suggested that homosynaptic LTP at PF–PC synapses is likely to be critical to mediate motor adaptation in vestibulo-ocular reflex (VOR) and delay eyeblink conditioning tasks [113]. First, it should be observed that even if LTD does not appear to be necessary for simple motor learning such as VOR and eyeblink conditioning, it remains unclear to what extent this result could be generalised to sensorimotor associative learning involved in spatial navigation. Second, as remarked by Schonewille et al. [112], it is not excluded that other cerebellar plasticity mechanisms may compensate for a deficient LTD at PF–PC synapses. Third, even if LTD had no functional implications in motor adaptation, the hypothesis of a cerebellar role in building internal models would remain plausible – although the mechanisms underlying the online shaping of built models would need to be redefined. Nevertheless, the current debate on the functional roles of LTD and LTP in cerebellar learning confirms the importance of including other plasticity sites in an extension of the presented model.

## Supporting Information

Figure S1
**Coding scheme for the inverse corrector implemented by the cerebellar microcomplex model.** Example of error encoding and output decoding for a positive correction of the right-side paws of the simulated mouse. The teaching signal encodes the angular error, that is the difference between desired 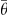 and actual 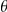 angular deviation. Here, the angular error 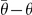 indicates that the velocity of right fore-and-hind paws must increase, i.e. 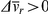. Then, the mean firing rate of IO neurons is set to 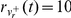 Hz, which makes LTD to take over LTP in the active PF–PC synapses of the corresponding microcomplex. The consequent decrease of PF–PC synaptic efficacy reduces the inhibitory action of PCs onto DCN neurons. Hence, the next time the microcomplex will receive the same contextual input, the average population activity of DCN neurons 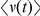 will increase, reinforcing the correction signal 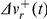.(EPS)

Figure S2
**Coding scheme for the forward predictor implemented by the cerebellar microcomplex model.** Example of error encoding and output decoding for the predicted rotation of the simulated mouse. The teaching signal encodes the actual rotation 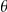 reached by the simulated animal after the execution of the last motor command. The firing rates of IO cells 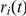 vary according to a set of radial basis functions spanning the 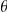 state space uniformly. A group of two IO cells share the same preferred angle and each group targets two distinct PCs, which in turn inhibit a single DCN unit. The latter codes for the same portion of the 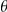 state space (and has the same preferred angle 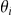) than the two IO cells that modulate its inhibitory PC afferents. Depending on the firing rate of a group of two IO cells, three cases must be distinguished and are shown in the figure: *(i)* if the firing rate of the two IO cells with preferred angle 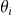 is 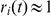 Hz (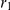 in this example), then LTD and LTP at PF–PC synapses of the two PCs driven by these two IO cells compensate each other and no learning occurs. The corresponding DCN neuron tends to stabilise its spike frequency (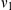); *(ii)* if the firing rate of the two IO cells with preferred angle 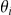 is 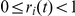 Hz (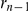 and 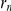 in this example), then LTP dominates and the corresponding DCN neuron tends to decrease its spike frequency (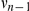 and 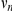); *(iii)* if the firing rate of the two IO cells with preferred angle 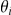 is 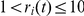 Hz (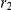 in this example), then LTD dominates LTP at the PF–PC synapses of the two PCs driven by these two IO cells. Thus, over training, the DCN unit whose preferred angle is close to 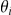 tends to increase its firing activity (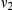 in this example). As a consequence, the decoding scheme used to readout the population activity of DCN neurons will tend towards an estimate of the next angular displacement 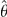 close to 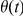. (The color code used to describe the intensity of neuronal discharges is the following: white for no activity, light blue for low activity, and dark blue for high activity).(EPS)

Figure S3
**Adaptation in forward predictor and inverse corrector cerebellar models.** **A.** Three examples of normalised prediction error for the angular position as a function of motor command presentations. **B.** Mean number of presentations – averaged over 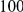 different motor commands – needed to create reliable context-response associations. Similar findings hold for the prediction of travelled distances (not shown). **C.** Time-course of forward predictor learning over the entire training in the MWM. Data points are averages over all animals (n = 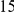 controls and n = 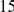 mutants) and all trials (n = 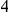) per day. Right y-axis: absolute number of learnt context-response associations. **D.** Mean prediction error for linear displacements. **E.** Mean prediction error for angular displacements. **F.** Three samples of residual normalised angular error as a function of number of desired state presentations. **G.** Mean normalised angular error averaged over 100 distinct desired states. **H.** Time course of inverse corrector performance gain of controls relative to mutants. The performance gain accounts for both distance and angular residual errors, and it is averaged over all animals and all trials of a day: 
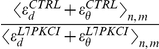
, with 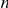 = 15 animals, 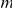 = 4 trials per day, 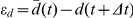, and 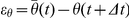. **I.** Mean residual translational error. **J.** Mean residual rotational error.(EPS)

Figure S4 **The hypothesis of a purely local procedural deficit in L7-PKCI mice does not account for all mutants' spatial navigation impairments observed experimentally.** Simulation results are shown in the main diagrams, whereas the corresponding experimental findigs are shown in the insets. **Results in the MWM:** **A.** Mean escape latency over training of simulated controls and mutants. **B.** Mean angular deviation between ideal and actual trajectory to the goal. **C.** Ratio between the time spent in the platform quadrant and the duration of a trial. **D.** Mean distance of the simulated mouse to the platform. **Results in the Starmaze:** **E.** Mean number of visited alleys. **F.** Mean distance swum in the Starmaze.
(EPS)

Figure S5 **Samples of navigation trajectories.** Examples of trajectories in the MWM for control (top) and mutant (bottom) simulated animals at different stages of training.
(EPS)

Figure S6 **The hypothesis of a global spatial behaviour deficit of L7-PKCI mice accounts for all mutants' navigation impairments observed experimentally.** **Results in the MWM:** **A.** Mean escape latency over training (left y-axis) and score (right y-axis) of simulated controls and mutants. **B.** Correlation between searching score and escape latency for control (top) and mutant (bottom) simulated mice. **C.** Mean angular deviation between ideal and actual trajectory to the goal. **D.** Ratio between the time spent in the platform quadrant and the total duration of a trial. **E.** Mean distance of the simulated mouse to the platform. **F.** Mean circling time. **Results in the Starmaze:** **G.** Mean number of visited alleys. **H.** Mean distance swum in the Starmaze.
(EPS)

Supplementary Methods S1 **Cerebellar microcomplex model.** This document provides equations and parameter settings related to the cerebellar microcomplex model, the connectivity layout, the coding scheme and the learning rules shaping the dynamics of the network.
(PDF)

Supplementary Methods S2 **Statistical analyses of neural activities.** This document provides a description of the set of statistical measures used to characterise the model neural code.
(PDF)

Supplementary Results S1 **Adaptation in forward and inverse cerebellar models.** This document provides our simulation results related to the adaptation performance of simulated forward and inverse models.
(PDF)

Supplementary Results S2 **Cerebellar role in local procedural spatial learning.** This document provides our results in a simulation where the procedural component of navigation is isolated, and demonstrates that a purely local sensorimotor adaptation deficit cannot account for navigation impairments observed experimentally in mutants.
(PDF)

Supplementary Results S3 **Cerebellar role in global tuning of spatial behaviour.** This document describes how differences between mutants and controls in the accuracy of the spatial code may result in differences in balancing exploration and exploitation behaviour.
(PDF)
