# Supplementary material for: Contribution of Cerebellar Sensorimotor Adaptation to Hippocampal Spatial Memory
Source: PLoS One. 2012 Apr 2;7(4):e32560. doi: 10.1371/journal.pone.0032560 (PMC3317659; doi:10.1371/journal.pone.0032560)
Supplement: Supplementary Methods S1 — Cerebellar microcomplex model. This document provides equations and parameter settings related to the cerebellar microcomplex model, the connectivity layout, the coding scheme and the learning rules shaping the dynamics of the network. (PDF) [file pone.0032560.s007.pdf]

Supplementary Methods S1

Cerebellar microcomplex model

Circuit model

The cerebellar microcomplex model (Fig. 1B in the main text) captures the main processing stages and connectivity layout (e.g. convergence/divergence ratios) of its biological counterpart (see [1] for a recent review). In the model, the MF layer consists of 100 cells' axons. MFs activate a population of 10^4 GCs, which produces a sparse representation of the input state space. Each MF is connected to a GC with a probability $P_{MF-GC} = 0.04$. Thus, each GC receives an average of 4 MF inputs and each MF projects onto approximately 400 GCs. GCs project via PFs to a population of 200 PCs. Each GC can create a connection to a PC with a probability $P_{GC-PC} = 0.75$. Thus, each PC can receive about 7500 input connections from PFs. MFs also excite a population of 100 DCN cells by all-to-all connections (P_{MF-DNC} = 1). In addition to the excitatory MF input, the discharge of each DCN unit is modulated by the inhibitory action of 2 afferent PCs. DCN activity constitutes the output of the cerebellar microcomplex model. A bidirectional long-term plasticity rule modifies the strength of PF–PC synapses, changing the input-output relationship of the circuit. The teaching signal for the learning rule is conveyed to the PCs by the population of 400 IO neurons, whose axons (i.e. CFs) project onto PCs through one-to-one connections. The highly simplified microcomplex model does not account for cerebellar interneurons, which are likely to be involved in denoising neurotransmission in the cerebellum [2], conveying timing information [3–5], and providing the biological substrate for the implementation of covariance-based learning rules [6, 7].

Different instances of the *same* microcomplex circuit are used to implement four inverse corrector models and two forward predictor models. The former learn to map desired positions onto motor commands to compensate for local drifts in swimming trajectories. Their inputs are the desired *next* distance $\bar{d}(t+\Delta t)$ and angle $\bar{\theta}(t+\Delta t)$, with $\Delta t = 200$ ms. Their outputs are the correction values $\Delta v_r, \Delta v_l$ for the speeds of the right-side and left-side paws of the simulated mouse. Two of the four inverse models learn to correct errors in the positive and negative ranges of left fore-and-hind paw speeds, $v_l^+(t)$ and $v_l^-(t)$, respectively. The other two learn to correct errors in the positive and negative ranges of right-side paw speeds, $v_r^+(t)$ and $v_r^-(t)$, respectively. Forward predictor models learn to infer next egocentric translations and orientations based on current motor commands. Their input is the motor command at time t, i.e. the pair of speeds (v_r, v_l) for the right-side and left-side paws. The output of the first forward model is the prediction $\hat{d}(t + \Delta t)$ of the distance travelled by the mouse at the end of the next time step. The output of the second forward predictor is the next egocentric orientation $\hat{\theta}(t + \Delta t)$ given the same motor command at time t.

Neuronal models

We model each MF as the axon of a leaky integrate-and-fire neuron whose membrane potential $V(t)$ dynamics is defined as:

$$C \frac{dV(t)}{dt} = g_{leak}\Big(V_{leak} - V(t)\Big) + I(t) \tag{S1}$$

where C denotes the membrane capacitance and g_{leak} the leak membrane conductance —i.e. $\tau = C/g_{leak}$ is the membrane time constant; V_{leak} is the resting membrane potential, and $I(t)$ the total synaptic drive. Whenever the membrane potential reaches a threshold V_{th} the neuron emits a spike. We used a time step $dt = 1$ ms for numerical integration.

Neuronal parameters					
		DCN	GR	PC	MF
V_{th}	mV	-60	-60	-60	-60
C	pF	2	2	2	2
g_{leak}	nS	2	2	2	2
V_{leak}	mV	-70	-70	-70	-70
g_{exc}	nS	0.1	0.2	60	-
V_{exc}	mV	0	0	0	-
g_{inh}	nS	2	-	-	-
V_{inh}	mV	-80	-80	-80	-
τ_{leak}	ms	20	20	20	20
τ_{exc}	ms	0.5	0.5	0.5	-
τ_{inh}	ms	10	-	-	-

Plasticity parameters		
Model	LTD (β)	LTP (α)
Forward	-0.5	1
Inverse	-0.025	0.1

Table S1. Parameter settings for neuronal and plasticity models.

GCs, PCs and DCN neurons are conductance based leaky integrate-and-fire units (similar to [8]) described by the following equation:

$$C\,\frac{dV(t)}{dt} = g_{leak}(t)\Big(V_{leak} - V(t)\Big) + g_{exc}(t)\Big(V_{exc} - V(t)\Big) + g_{inh}(t)\Big(V_{inh} - V(t)\Big) \tag{S2}$$

where the membrane potential $V(t)$ depends on an excitatory synaptic conductance g_{exc}, an inhibitory conductance g_{inh}, and a leaky conductance g_{leak}. V_{leak}, V_{exc}, and V_{inh} are the corresponding resting potentials. Again, when $V(t)$ reaches a threshold V_{th} the cell emits a spike. All active conductances $g_{leak}(t)$, $g_{exc}(t)$, $g_{inh}(t)$ vary according to:

$$g(t) = g\sum_j W_j \int_{-\infty}^{t} \exp\Big(\frac{t-t'}{\tau}\Big)\delta(t-t')dt \tag{S3}$$

where g is the maximal conductance, W_j is the efficacy of the projection from presynaptic neuron j (with hard bounds $[0, 1]$), τ is the synaptic time constant, t' is the time of a presynaptic spike, and $\delta(t - t')$ is a Dirac function equal to 1 only when the presynaptic neuron emits a spike at time t'.

The discharge of each IO neuron is modelled by a discrete Poisson spike-train generator with rate $r(t)$ determined by the corresponding teaching signal (see below).

Table S1 provides the parameter settings for all neuronal models used in our simulations.

Encoding MF cerebellar inputs

MFs constitute the main input stage of the cerebellar microcomplex [9]. In the model, they carry information about desired future states (for the input of inverse corrector microcomplexes) and motor commands (for the input of forward predictor microcomplexes). A future state consists of a pair $(\bar{d}, \bar{\theta})$ of desired distance and rotation at time $t + \Delta t$, whereas a motor command consists of a pair of speeds (v_r, v_l) to be applied at time t. For each input variable (e.g. the next desired distance $\bar{d}$), a population

of 50 MFs acts as a family of radial basis functions encoding the values of a variable. More specifically, the input current $I_i(t)$ (Eq. S1) of each MF neuron i is:

$$I_i(t) = \gamma + \exp\Big(- \frac{(x(t) - \mu_i)^2}{2\sigma^2}\Big) \tag{S4}$$

where μ_i and σ^2 are the centre and the variance of the kernel associated to the MF i, respectively. The distribution of all kernel centres μ_i covers the input space uniformly and the variance parameter σ^2 ensures a small overlap between MF responses. The γ constant factor in Eq. S4 endows each MF neuron with intrinsic spontaneous activity at about 5 Hz, whereas the parameters regulating the discharge of MFs (Eq. S1) limit their activity to 50 Hz.

Decoding DCN cerebellar outputs

Decoding DCN activity in inverse corrector models

For the four inverse corrector models, the decoding of DCN activity must produce motor command adjustments —i.e. positive and negative right-side speeds ($\Delta v_r^+(t)$, $\Delta v_r^-(t)$) and left-side speeds ($\Delta v_l^+(t)$, $\Delta v_l^-(t)$). For each of the four inverse models, an average decoding scheme maps DCN outputs into speed corrections. For instance, as shown in Figure S1 for the microcomplex correcting positive right paw velocities, we take:

$$\Delta v_r^+(t) = \frac{A}{\nu_{max}} \cdot \Big\langle \nu_i(t) \Big\rangle_{i \in DCN} \tag{S5}$$

where $\nu_i(t)$ denotes the instantaneous spike frequency of a DCN neuron i, calculated by averaging over a rectangular sliding window of 50 ms; the normalisation term $\nu_{max} = 200$ Hz is the maximum spike frequency of DCN cells; the scaling factor A determines the maximum correction amplitude. Similarly, the output of the microcomplex devoted to negative right velocities produces a decoded signal $\Delta v_r^-(t)$. Then, the overall correction for the right fore-and-hind paws is:

$$\Delta v_r(t) = \Delta v_r^+(t) + \Delta v_r^-(t) \tag{S6}$$

A similar decoding scheme maps the outputs of the two microcomplexes correcting left fore-and-hind paw velocities into a signal $\Delta v_l(t)$.

Decoding DCN activity in forward predictor models

For the two forward predictor models, the decoding of DCN activity at time t must predict the travelled distance $\hat{d}$ and the rotation $\hat{\theta}$ of the animal at time $t + \Delta t$. A population decoding scheme computes the predictions $\hat{d}(t)$ and $\hat{\theta}(t)$ based on the DCN activity (see example in Fig. S2):

$$\hat{d}(t) = \frac{\sum_i \nu_i(t) \cdot d_i}{\sum_i \nu_i(t)} \tag{S7}$$

$$\hat{\theta}(t) = \frac{\sum_i \nu_i(t) \cdot \theta_i}{\sum_i \nu_i(t)} \tag{S8}$$

where $\nu_i(t)$ indicates the instantaneous spike frequency of a DCN neuron i, d_i denotes the preferred distance encoded by a DCN neuron i in the first forward model, and θ_i denotes the preferred angular displacement of a DCN neuron i in the second forward model —positive angles are defined counter-clockwise. Both preferred distance and angle values are evenly distributed over the output space (Fig. S2).

Synaptic efficacy and plasticity rules

Only the PF–PC synapses of the simulated microcomplex circuit are plastic —i.e. the model does not account for other plasticity sites in the real cerebellar microcomplex [10–13].

The tuning of non-plastic synapses and neuronal parameters is such that the activity of model cerebellar units is compatible with experimental data. The discharge of simulated GCs is consistent with recent *in vivo* recordings suggesting that joint-related movement inputs generate sustained GC activity at ~ 150 Hz and that two or more MF input spikes are necessary to elicit one GC burst of spikes [14–17]. Simple spikes of model PCs occur at a frequency ≤ 150 Hz [18] when PCs are activated by PFs. Complex spikes of simulated PCs, caused by a single discharge of the afferent CF, correspond to learning triggering events —we do not simulate high frequency components of the bursts. Finally, DCN neurons discharge at mean firing rates of ~ 20 Hz in the presence of active PC inhibitory inputs [19], whereas their activity upper-bound is 200 Hz otherwise.

Model PF–PC synapses undergo bidirectional long-term plasticity, i.e. both potentiation, LTP, and depression, LTD. We implement LTP at each PF–PC synapse as a non-associative weight increase triggered by each GC spike, consistent with the homosynaptic rule described by Lev-Ram et al. [20]:

$$\Delta W^{+}_{PF-PC}(t) = \alpha \cdot \delta(t - t_{GC}) \tag{S9}$$

where α denotes the gain factor for LTP. The weight is increased only when the presynaptic GC emits a spike at time $t = t_{GC}$.

We implement LTD at PF–PC synapses as an associative weight decrease triggered by a spike from the IO. This is in agreement with the heterosynaptic plasticity mechanism described by Ito et al. [21]:

$$\Delta W^{-}_{PF-PC}(t) = -\beta \cdot \int_{-\infty}^{t_{IO}} f(t - t_{GC})\, \delta(t - t_{IO})\, dt \tag{S10}$$

where β is a gain factor for LTD, and the temporal kernel function f correlates each IO spike with the past discharge of a GC [8]. In the model, the largest LTD amplitude occurs when the PC receives an IO spike approximately 100 ms after an input spike from a GC, consistent with Safo et al. [22].

The total change in synaptic weight in one PF–PC synapse is then:

$$\Delta W_{PF-PC}(t) = \Delta W^{+}_{PF-PC}(t) + \Delta W^{-}_{PF-PC}(t) \tag{S11}$$

Table S1 provides the parameter settings for the implemented plasticity models.

Teaching signals

In both inverse corrector and forward predictor models, IO neurons convey teaching signals via the CFs that target PCs and mediate LTD at PF–PC synapses.

Teaching signal for the inverse corrector model

For the four inverse models, the teaching signal encodes the difference between desired states $\bar{d}$, $\bar{\theta}$ and actual states d, θ. After the execution of each motor command, the translational error $\varepsilon_d = \bar{d} - d$ and the angular error $\varepsilon_\theta = \bar{\theta} - \theta$ indicate the direction of change of the speed of left- and right-side paws:

$$\begin{aligned}
\varepsilon_d > 0,\ \varepsilon_\theta > 0 \quad &\Longrightarrow \bar{v}_r > v_r \quad \Longrightarrow \Delta\bar{v}_r > 0\\
\varepsilon_d < 0,\ \varepsilon_\theta < 0 \quad &\Longrightarrow \bar{v}_r < v_r \quad \Longrightarrow \Delta\bar{v}_r < 0\\
\varepsilon_d > 0,\ \varepsilon_\theta < 0 \quad &\Longrightarrow \bar{v}_l > v_l \quad \Longrightarrow \Delta\bar{v}_l > 0\\
\varepsilon_d < 0,\ \varepsilon_\theta > 0 \quad &\Longrightarrow \bar{v}_l < v_l \quad \Longrightarrow \Delta\bar{v}_l < 0
\end{aligned}$$

where $\bar{v}_x$ denotes the desired speed, and $\Delta\overline{v}_x$ denotes the speed error signal. The speed error signal determines the activity of IO neurons, which in turn modulates synaptic plasticity at PF–PC synapses. The mean Poisson firing rates $r_{v_r^+}(t)$ and $r_{v_r^-}(t)$ of IO neurons in the two microcomplexes for positive and negative right-side velocities, respectively, vary according to:

$$r_{v_r^+}(t) = k \cdot \mathcal{H}(\Delta\overline{v}_r) \quad \text{(S12)}$$

$$r_{v_r^-}(t) = k \cdot \mathcal{H}(-\Delta\overline{v}_r) \quad \text{(S13)}$$

where $k = 10$ is a scaling factor and $\mathcal{H}$ is the Heaviside function defined such that $\mathcal{H}(0) = 0.1$. According to Eqs. S12, S13 (see also example in Fig. S1):

- When no speed change is needed, i.e. $\Delta\overline{v}_r = 0$, then the mean IO firing rates are $r_{v_r^+}(t) = r_{v_r^-}(t) = 1$ Hz, which make heterosynaptic LTD (Eq. S10) and homosynaptic LTP (Eq. S9) at PF–PC synapses to compensate each other —i.e. no adaptation occurs.
- When the velocity of right fore-and-hind paws must increase, i.e. $\Delta\overline{v}_r > 0$, then $r_{v_r^+}(t) = 10$ Hz, which makes LTD to take over LTP in the active PF–PC synapses of the corresponding microcomplex. The consequent decrease of PF–PC synaptic efficacy reduces the inhibitory action of PCs onto DCN neurons the next time that the microcomplex receives the same contextual input —which activates the same PF–PC synapses and then the same PC responses. As a consequence, the population activity of DCN neurons increases, which reinforces the correction signal $\Delta v_r^+(t)$ (Eq. S5). In addition, for $\Delta\overline{v}_r > 0$, the mean IO firing rates $r_{v_r^-}(t) = 0$ Hz, which blocks LTD in the active PF–PC synapses of the corresponding microcomplex. Thus, LTP increases and strengthens future inhibitory actions of PCs onto DCN neurons in the presence of the same contextual input to the microcomplex. Then, the corrective signal $\Delta v_r^-(t)$ decreases over time. As a consequence, the resultant correction $\Delta v_r(t)$ (Eq. S6) tends to increase and become positive over training.
- Conversely, when the velocity of right paws must decrease, i.e. $\Delta\overline{v}_r < 0$, the overall correction $\Delta v_r(t)$ tends to decrease and become negative over training.

The modulation of the firing rates $r_{v_l^+}(t)$ and $r_{v_l^-}(t)$ of IO neurons in the two microcomplexes correcting left-side speeds is computed similarly.

Teaching signal for the forward predictor model

For the two forward predictors, the teaching signals encode the actual distance d and rotation θ reached by the simulated animal after execution of the last motor command. As shown in Figure S2, the mean firing rates of IO cells in the microcomplex subserving angular change predictions vary according to a set of radial basis functions spanning the θ state space uniformly. That is, the mean firing rate $r_i(t)$ of a Poisson IO neuron i is taken as:

$$r_i(t) = k \cdot \exp\Big(- \frac{(\theta(t) - \theta_i)^2}{2\sigma_i^2} \Big) \quad \text{(S14)}$$

where $k = 10$ is a scaling factor, θ_i is the "preferred angle" of the cell, and σ determines the degree of overlap between adjacent IO responses. In this microcomplex, groups of two IO cells share the same preferred angles (Fig. S2). Each group of two IO neurons targets two distinct PCs, which in turn inhibit the same DCN unit. The latter codes for the same portion of the θ state space (and has the same preferred angle θ_i) than the two IO cells that modulate its inhibitory PC afferents.

According to Eq. S14 and to the plasticity rules described above:

- If the firing rate of the two IO cells with preferred angle θ_i is $r_i(t) \approx 1$ Hz, then LTD and LTP at PF–PC synapses of the two PCs driven by these two IO cells compensate each other and no learning occurs.

- If the firing rate of the two IO cells with preferred angle θ_i is $1 < r_i(t) \leq 10$ Hz, then LTD dominates LTP at the PF–PC synapses of the two PCs driven by these two IO cells. Thus, over training, the DCN unit whose preferred angle is close to θ_i tends to increase its firing activity, whereas the other DCN units tend to either decrease or maintain their spike frequency. As a consequence, the decoding scheme used to readout the population activity of DCN neurons in the forward predictor model (Eq. S8) will tend towards an estimate of the next angular displacement close to θ_i.
- Conversely, if the firing rate of the two IO cells with preferred angle θ_i is $0 \leq r_i(t) < 1$ Hz, then LTP dominates and the corresponding DCN neuron tends to decrease its spike frequency. Thus, this DCN unit will not contribute to population coding significantly.

Bistability of the forward model

In line with previous hypotheses [23], we assumed that forward predictions are useful only when accurate. The accuracy of the forward prediction is reflected by a bistable behaviour, with active DCN neurons in the presence of reliable predictions and with below-threshold DCN activity otherwise. This all-or-none response results from the connectivity and coding schemes employed for the forward model. Recall that each DCN unit and each IO cell codes for a specific value of a variable, with the whole range of values being distributed over the entire population of cells —which acts as a family of radial basis functions.

The following example illustrates how the all-or-none response of the forward model works. Before training, DCN neurons discharge at baseline activity due to dominant PC inhibition —which results from the initialised large synaptic weights between PFs and PCs. When feeding this "naïve" network with a motor command M, the latter activates a subset of granular cells G, which in turn excite the PCs and then inhibit DCN activity. Let us now suppose that the actual execution of M produces a rotation θ, and let IO_θ be the subset of IO cells coding for that rotation value. The discharge of IO_θ cells triggers LTD between active PFs and PCs. Let PC_θ denote the subgroup of PCs targeted by IO_θ. The induction of LTD decreases the connection strength between G and the PC_θ. Then, the activity of PC_θ directly influences the activity of the subgroup of DCN cells that code for the angular rotation θ, i.e. DCN_θ. Reiterating this adaptation process will incrementally decrease the activity of the PC_θ population and prevent it from inhibit DCN_θ cells completely. Hence, in response to the next presentation of the motor command M, DCN_θ cells will discharge and the output of the forward model will code for the predicted angular deviation θ. Conversely, if we now present a new motor command M', a new set G' of granule cells will become active. Since previous training did not modify the connections between G' and PCs, DCN cells will remain silent (due to dominant PC inhibition).

References

1. Ito M (2006) Cerebellar circuitry as a neuronal machine. Prog Neurobiol 78: 272–303.
2. Hirano T, Watanabe D, Kawaguchi SY, Pastan I, Nakanishi S (2002) Roles of inhibitory interneurons in the cerebellar cortex. Ann N Y Acad Sci 978: 405–412.
3. Desmond JE, Moore JW (1988) Adaptive timing in neural networks: the conditioned response. Biol Cybern 58: 405–415.
4. Yamazaki T, Tanaka S (2007) The cerebellum as a liquid state machine. Neural Netw 20: 290–297.
5. D'Angelo E, De Zeeuw CI (2009) Timing and plasticity in the cerebellum: focus on the granular layer. Trends Neurosci 32: 30–40.
6. Sejnowski TJ (1977) Storing covariance with nonlinearly interacting neurons. J Math Biol 4: 303–321.

7. Dean P, Porrill J, Ekerot CF, Jörntell H (2010) The cerebellar microcircuit as an adaptive filter: experimental and computational evidence. Nat Rev Neurosci 11: 30–43.

8. Carrillo RR, Ros E, Boucheny C, Coenen OJMD (2008) A real-time spiking cerebellum model for learning robot control. Biosystems 94: 18–27.

9. Eccles JC, Ito M, Szentágothai J (1967) The Cerebellum as a Neuronal Machine. Springer-Verlag.

10. Hansel C, Linden DJ, D'Angelo E (2001) Beyond parallel fiber LTD: the diversity of synaptic and non-synaptic plasticity in the cerebellum. Nat Neurosci 4: 467–475.

11. Boyden ES, Katoh A, Raymond JL (2004) Cerebellum-dependent learning: the role of multiple plasticity mechanisms. Annu Rev Neurosci 27: 581–609.

12. De Zeeuw CI, Yeo CH (2005) Time and tide in cerebellar memory formation. Curr Opin Neurobiol 15: 667–674.

13. Pugh JR, Raman IM (2009) Nothing can be coincidence: synaptic inhibition and plasticity in the cerebellar nuclei. Trends Neurosci 32: 170–177.

14. Chadderton P, Margrie TW, Häusser M (2004) Integration of quanta in cerebellar granule cells during sensory processing. Nature 428: 856–860.

15. Jörntell H, Ekerot CF (2006) Properties of somatosensory synaptic integration in cerebellar granule cells in vivo. J Neurosci 26: 11786–11797.

16. Rancz EA, Ishikawa T, Duguid IC, Chadderton P, Mahon S, et al. (2007) High-fidelity transmission of sensory information by single cerebellar mossy fibre boutons. Nature 450: 1245–1248.

17. Arenz A, Silver RA, Schaefer AT, Margrie TW (2008) The contribution of single synapses to sensory representation in vivo. Science 321: 977–980.

18. Raman IM, Bean BP (1999) Properties of sodium currents and action potential firing in isolated cerebellar Purkinje neurons. Ann N Y Acad Sci 868: 93–96.

19. Lamont M (2009) An electrophysiological analysis of deep cerebellar nuclei, with particular focus on Kv3 channels. Bioscience Horizons 2: 55–63.

20. Lev-Ram V, Wong ST, Storm DR, Tsien RY (2002) A new form of cerebellar long-term potentiation is postsynaptic and depends on nitric oxide but not cAMP. Proc Natl Acad Sci U S A 99: 8389–8393.

21. Ito M, Kano M (1982) Long-lasting depression of parallel fiber-Purkinje cell transmission induced by conjunctive stimulation of parallel fibers and climbing fibers in the cerebellar cortex. Neurosci Lett 33: 253–258.

22. Safo P, Regehr WG (2008) Timing dependence of the induction of cerebellar LTD. Neuropharmacology 54: 213–218.

23. Shadmehr R, Smith MA, Krakauer JW (2010) Error correction, sensory prediction, and adaptation in motor control. Annu Rev Neurosci 33: 89–108.
