# Supplementary material for: Contribution of Cerebellar Sensorimotor Adaptation to Hippocampal Spatial Memory
Source: PLoS One. 2012 Apr 2;7(4):e32560. doi: 10.1371/journal.pone.0032560 (PMC3317659; doi:10.1371/journal.pone.0032560)
Supplement: Supplementary Methods S2 — Statistical analyses of neural activities. This document provides a description of the set of statistical measures used to characterise the model neural code. (PDF) [file pone.0032560.s008.pdf]

Supplementary Methods S2

Statistical analyses of neural activities

In order to quantify spatial-related correlates of neural activity, we discretise the continuous two-dimensional input space by a grid of square pixels (5×5 cm). Let S denote the set of stimuli —i.e. the set of pixels s visited by the simulated animal while solving the task.

Place field area. For each neuron, we compute the mean firing rate associated to each pixel s by dividing the spike count associated to s by the time spent by animal at location s. We then estimate the size of a receptive (place) field as the number of adjacent pixels with a firing rate above the grand mean rate —i.e. total spike count divided by the total time spent moving in the maze— plus the standard deviation [1, 2].

Spatial coherence. We assess the local smoothness of place fields as the z-transform of the correlation between the firing rate in each element of the positional firing rate array and the aggregate rate in the eight nearest pixels [2].

Multimodality and number of peaks of receptive fields. We assess the statistical significance of the unimodality *vs.* multimodality property of spatial receptive fields by means of the Hartigan DIP test [3]. We estimate the number of peaks of a receptive field as the number of distinct activity blobs with a firing rate above the grand mean rate plus standard deviation.

Spatial density of receptive fields. To assess the redundancy level of a spatial code —i.e. the average number of units encoding a spatial location $s \in S$— we use the following density measure:

$$D_S = \left\langle \sum_{j \in J} \mathcal{H}\Big(r_j(s) - \eta\Big) \right\rangle_{s \in S} \tag{S1}$$

where $r_j(s)$ is the response of a neuron $j \in J$ when the animal is visiting the location $s \in S$, η denotes the noise level activity, and $\mathcal{H}$ is the Heaviside function.

Spatial information content of the spatial code. We quantify how much information is conveyed by neural responses $r \in R$ about spatial locations $s \in S$ by computing Shannon mutual information I$(R; S)$ [4, 5]:

$$I(R;S) = \sum_{s \in S} p(s) \sum_{r \in R} p(r|s) \cdot \log_2 \left(\frac{p(r|s)}{p(r)} \right) = \sum_{s \in S} p(s) \cdot I(R;s) \tag{S2}$$

where $p(r|s)$ indicates the conditional probability of recording a response r while having the simulated rat visiting a region s; $p(s)$ the a priori probability computed as the ratio between time spent at place s and the total time; $p(r) = \sum_{s \in S} p(s) \cdot p(r|s)$ the marginal probability of observing a neural response r; and $I(R; s)$ is the *stimulus-specific surprise* [6]. We discretise the continuous output space of a neuron $R = [0, 1]$ by means of a binning procedure (bin-width equal to 0.1). We subtract a correcting term C to mutual information to limit the sampling bias [7]:

$$C = \frac{\sum_s R_s^+ - R^+ - (|S| - 1)}{2N\, ln(2)} \tag{S3}$$

where $R_s^+ = \sum_{r \in R} \mathcal{H}(p(r|s))$ denotes the number of response bins in which the occupancy probability $p(r|s) > 0$; $R^+ = \sum_{r \in R} \mathcal{H}(p(r))$ denotes the number of response bins where $p(r) > 0$; $|S|$ is the number of stimuli; N is the number of stimulus-response pairs (s, r).

We compute the mutual information of both single unit responses, i.e. $I_i(R; S)$, and population responses, i.e. $I_{pop}(R; S)$. The ratio:

$$I^*(R; S) = \frac{I_{pop}(R; S)}{\sum_i I_i(R; S)} \tag{S4}$$

measures the "information sparseness" of a population code, or, conversely, the redundancy level of the spatial information content of a neural code.

Mutual information quantifies the mean information content over the whole environment, but it does not quantifies the specificity of the neuronal discharges. Thus, we employ an additional measure, namely the information per spike I_{spike} [8] defined for a neuron j as:

$$I_{spike}(j) = \sum_{s \in S} \frac{r_j(s)}{\bar{r}_j} \cdot \log_2 \left(\frac{r_j(s)}{\bar{r}_j} \right) \cdot p(s) \tag{S5}$$

where $r_j(s)$ is the activity of neuron j at position s and $\bar{r}_j$ is the overall mean activity of neuron j.

Accuracy of the population space code. To assess the quality of a hippocampal place map, we measure the mean accuracy of the population vector estimate [9, 10]. Let $r_i(s)$ denote the activity of a neuron i at position s, and s_i be the centre of its place field. The population vector $\tilde{s}$ is the centre of mass of the ensemble activity:

$$\tilde{s} = \frac{\sum_i r_i(s) \cdot s_i}{\sum_i r_i(s)} \tag{S6}$$

The mean localisation error is simply taken as the mean Euclidean distance between actual positions s and estimates $\tilde{s}$:

$$\varepsilon = \langle \varepsilon(s) \rangle_{s \in S'} = \left\langle \sqrt{\sum_n (\tilde{s}_n - s_n)^2} \right\rangle_{s \in S'} \tag{S7}$$

where $S' \subseteq S$ is the set of positions where $\tilde{s}$ is well defined (i.e. $\sum_i r_i(s) \neq 0$).

Percentage of recognised locations. We measure the fraction of the environment encoded with high accuracy by the population activity of simulated place cells as:

$$R = \left\langle \mathcal{H}\Big(\xi - \varepsilon(s) \Big) \right\rangle_{s \in S} \tag{S8}$$

where $\varepsilon(s)$ is the place recognition error when the animal is actually visiting the location $s \in S$, $\xi = 10$ cm is the error threshold below which the estimate is considered accurate, and $\mathcal{H}$ is the Heaviside function.

References

1. Muller RU, Kubie JL, Ranck B (1987) Spatial firing patterns of hippocampal complex-spike cells in a fixed environment. Cell 7: 1935–1950.

2. Hok V, Save E, Lenck-Santini PP, Poucet B (2005) Coding for spatial goals in the prelimbic/infralimbic area of the rat frontal cortex. Proc Natl Acad Sci U S A 102: 4602–4607.

3. Hartigan JA, Hartigan PM (1985) The Dip test of unimodality. Ann Stat 13: 70–84.

4. Shannon C (1948) A mathematical theory of communication. Bell Syst Tech J 27: 379–423.
5. Bialek W, Rieke F, De Ruyter Van Steveninck RR, Warland D (1991) Reading a neural code. Science 252: 1854–1857.
6. DeWeese MR, Meister M (1999) How to measure the information gained from one symbol. Network 10: 325–340.
7. Panzeri S, Treves A (1996) Analytical estimates of limited sampling biases in different information measures. Network 7: 87–107.
8. Skaggs WE, McNaughton BL, Gothard KM, Markus EJ (1993) An information-theoretic approach to deciphering the hippocampal code. In: Hanson SJ, Cowan JD, Giles LC, editors, Neural Inf Process Syst. volume 1990 (5), pp. 1030–1037.
9. Georgopoulos AP, Schwartz AB, Kettner RE (1986) Neuronal population coding of movement direction. Science 233: 1416–1419.
10. Wilson MA, McNaughton BL (1993) Dynamics of the hippocampal ensemble code for space. Science 261: 1055–1058.
