# Supplementary material for: Contribution of Cerebellar Sensorimotor Adaptation to Hippocampal Spatial Memory
Source: PLoS One. 2012 Apr 2;7(4):e32560. doi: 10.1371/journal.pone.0032560 (PMC3317659; doi:10.1371/journal.pone.0032560)
Supplement: Supplementary Results S1 — Adaptation in forward and inverse cerebellar models. This document provides our simulation results related to the adaptation performance of simulated forward and inverse models. (PDF) [file pone.0032560.s009.pdf]

Supplementary Results S1

Adaptation in forward and inverse cerebellar models

We tested the adaptation performance of forward and inverse models by training them during navigation in the Morris water maze (MWM). We simulated a group of control mice ($n = 15$) and a group of mutants ($n = 15$) —i.e. with disabled LTD in PF–PC synapses.

Learning performance of forward predictor models

The prediction error of forward cerebellar models was large at the beginning of training, but it decreased to small values after few motor command presentations (Fig. S3 A). The mean number of presentations necessary to learn reliable predictions was 7.5 ± 0.5 (mean $\pm$ s.e.) in simulated controls (Fig. S3 B, white bar). Expectedly, DCN units of simulated mutants remained inhibited over an infinite number of presentations (Fig. S3 B, black bar), since no learning occurred.

The learning time course over an entire training session in the MWM (Fig. S3 C) shows that, after day 1, simulated controls could reliably predict the outcome of $\sim 30\%$ of motor commands —i.e. they could estimate both future rotation angles $\theta(t + \Delta t)$ and travel distances $d(t + \Delta t)$ prior to the execution of 30% of the received commands. The performance of the forward predictors increased monotonically and reached $65 - 70\%$ at the end of training. This corresponded to an absolute number of ~ 300 distinct motor commands that were reliably predicted by the forward model at day $9 - 10$ (Fig. S3 C, right y-axis). The mean prediction errors were 2.8 ± 0.2 mm and 6 ± 0.6 degrees for distances and rotations, respectively (Figs. S3 D, E). As expected, simulated mutants did not learn any sensorimotor association (Fig. S3 C, D, E).

Learning performance of inverse corrector models

The residual error of inverse correctors (i.e. difference between desired and actual state after movement correction) decreased steadily over time but significantly slower compared to forward learning (Fig. S3 F). Indeed, the convergence of the learning process required ~ 100 presentations for inverse corrector models to bound post-execution errors effectively (Fig. S3 G).

The relative gain in terms of learning performance of controls *vs.* mutants increased monotonically over the entire training in the MWM and it reached $\sim 135 - 140\%$ after $9 - 10$ days (Fig. S3 H). The mean distance error over all training trials was significantly smaller in controls than mutants (ANOVA, $F_{1,28} = 63.92$, $P < 0.001$) and reached 16.9 ± 0.8 mm *vs.* 23.8 ± 0.8 mm (Fig. S3 I). Similarly, after each movement execution, controls corrected significantly better their orientation than mutants (ANOVA, $F_{1,28} = 64.01$, $P < 0.001$), with an error of about 12.2 ± 0.9 *vs.* 22.0 ± 0.9 degrees (Fig. S3 J).
