# Supplementary material for: Contribution of Cerebellar Sensorimotor Adaptation to Hippocampal Spatial Memory
Source: PLoS One. 2012 Apr 2;7(4):e32560. doi: 10.1371/journal.pone.0032560 (PMC3317659; doi:10.1371/journal.pone.0032560)
Supplement: Supplementary Results S2 — Cerebellar role in local procedural spatial learning. This document provides our results in a simulation where the procedural component of navigation is isolated, and demonstrates that a purely local sensorimotor adaptation deficit cannot account for navigation impairments observed experimentally in mutants. (PDF) [file pone.0032560.s010.pdf]

Supplementary Results S2

Cerebellar role in *local* procedural spatial learning

Can a purely local motor adaptation impairment account for all observed spatial navigation deficits of L7-PKCI mice? To test this hypothesis, we isolated the procedural component of navigation by blocking the functional projection from the cerebellar forward predictor to the hippocampal network (red projection in Fig. 1A). That is, we assumed that simulated control and L7-PKCI mice would have equivalent spatial representation capabilities.

In the MWM, the escape latency of simulated mutants was significantly larger compared to controls over the entire training period (Fig. S4 A, ANOVA, $F_{1,28} = 18.92$, $P < 0.001$). Similarly, simulated L7-PKCI were significantly impaired in reducing the (local) angular deviation between actual and ideal trajectory to the platform (Fig. S4 B, ANOVA, $F_{1,28} = 23.57$, $P < 0.001$). These differences between simulated controls and mutants were not due to differences in swimming speeds (ANOVA, $F_{1,28} = 2.5$, $P > 0.05$; data not shown). Importantly, both controls and mutants improved their goal-directed navigation over training, with a tendency to stabilise their performances after day 7 (Figs. S4 A, B). Hence, simulated L7-PKCI mice could solve the MWM task, although their spatial behaviour was significantly suboptimal compared to controls. These simulation results are qualitatively consistent with experimental findings by Burguière et al. [1] (insets of Figs. S4 A, B). However, the mean intergroup differences for both behavioural parameters —e.g. for the heading ϕ, we computed $\left\langle \phi^{CTRL} - \phi^{L7PKCI} \right\rangle_{n,m}$ by averaging over all $n = 15$ animals and all $m = 40$ training trials— were significantly smaller in simulations compared to experimental data (for escape latency: ANOVA, $F_{1,28} = 10.93$, $P < 0.001$; for heading: ANOVA, $F_{1,28} = 14.65$, $P < 0.001$).

In contrast to experimental observations, the goal-searching behaviour of simulated controls and mutants was not significantly different (Fig. S4 C). Indeed, the ratio between the time spent within the platform quadrant and the trial duration increased over training for both simulated groups with no significant intergroup differences (ANOVA, $F_{1,28} = 3.1$, $P > 0.05$). Similarly, the mean distance-to-goal was not significantly larger in simulated mutants compared to controls (Fig. S4 D; ANOVA, $F_{1,28} = 3.7$, $P > 0.05$). Note that the purely local procedural hypothesis merely assumed a deficit of cerebellar-dependent *fine tuning* of navigation trajectories in L7-PKCI mice —due to the impaired inverse corrector and forward predictor learning in simulated mutants. See Fig. S5 for some examples of suboptimal trajectories in simulated mutants (compared to controls) caused by a lack of local minimisation of motor-command execution errors. On the other hand, under this hypothesis there was no built-in deficit that might possibly account for a less efficient goal-searching spatial behaviour in simulated L7-PKCI mice.

In the Starmaze task, simulated mutants and controls exhibited comparable performance over training in terms of number of visited alleys (Fig. S4 E; ANOVA $F_{1,18} = 0.19$, $P > 0.5$), mean distance swum to reach the target (Fig. S4 F; ANOVA $F_{1,18} = 0.20$, $P > 0.5$), escape latency (not shown, ANOVA $F_{1,18} = 1.11$, $P > 0.25$), and heading-to-goal (not shown, ANOVA $F_{1,18} = 2.01$, $P > 0.1$). These findings are consistent with experimental observations (insets of Figs. S4 E,F).

References

1. Burguière E, Arleo A, Hojjati M, Elgersma Y, De Zeeuw CI, et al. (2005) Spatial navigation impairment in mice lacking cerebellar LTD: a motor adaptation deficit? Nat Neurosci 8: 1292–1294.
