# Supplementary material for: Contribution of Cerebellar Sensorimotor Adaptation to Hippocampal Spatial Memory
Source: PLoS One. 2012 Apr 2;7(4):e32560. doi: 10.1371/journal.pone.0032560 (PMC3317659; doi:10.1371/journal.pone.0032560)
Supplement: Supplementary Results S3 — Cerebellar role in global tuning of spatial behaviour. This document describes how differences between mutants and controls in the accuracy of the spatial code may result in differences in balancing exploration and exploitation behaviour. (PDF) [file pone.0032560.s011.pdf]

Supplementary Results S3

Cerebellar role in *global tuning* of spatial behaviour

Our results suggested a joint effect of low-level motor adaptation deficits and (hippocampal-dependent) spatial learning impairments in L7-PKCI mice (Sec. 3.1 and Supplementary Results S2). As a consequence, cerebellar sensorimotor adaptation may play a role in high-level components of navigation, by contributing to the global tuning of spatial behaviour. We tested the hypothesis of an impact of cerebellar learning deficits on the ability of L7-PKCI mice to balance their exploration-exploitation trade-off. We isolated this possible effect by blocking the functional interaction between cerebellum and hippocampus (Fig. 1 A), and by artificially unbalancing the exploration-exploitation control in mutants. To do so, we increased the exploration probability of mutants over the entire training period compared to controls —by taking $P_{explore}^{L7-PKCI} = 0.25$ *vs.* $P_{explore}^{CTRL} = 0.1$. We also artificially impaired the ability of mutants to inhibit thigmotaxic behaviour by biasing their P_{circ} function (Eq. 1) such that L7-PKCI mice were likely to perform peripheral circling over a larger number of training trials than controls.

In the MWM, both simulated groups improved their goal navigation performances over training (Fig. S6). However, the escape latency of L7-PKCIs decreased significantly slower and to a smaller extent than in controls over time (Fig. S6 A, ANOVA, $F_{1,28} = 68.69$, $P < 0.001$). Also, the search scores were larger in simulated mutants than in controls over training (Fig. S6 A, right y-axis). As already reported by Burguière et al. [1], search scores were highly correlated to escape latencies in both controls and mutants (Fig. S6 B) —confirming that the longer time-to-goal needed by L7-PKCI mice was linked to a suboptimal searching behaviour. A difference of swimming speed was not responsible for these intergroup differences (not shown, ANOVA, $F_{1,28} = 1.5385$, $P > 0.1$). The heading-to-goal measure suggested a significant deficit of simulated mutants in optimally orienting their movements toward the hidden platform (Fig. S6 C, ANOVA, $F_{1,28} = 32.26$, $P < 0.001$). The ratio between the time spent within the platform quadrant and the trial duration was significantly larger in controls over the entire training (Fig. S6 D, ANOVA, $F_{1,28} = 44.16$, $P < 0.001$). Also, the mouse-to-platform distance measure suggested that mutants tended to follow significantly longer goal pathways than controls (Fig. S6 E, ANOVA, $F_{1,28} = 63.723$, $P < 0.001$). Expectedly, mutants exhibited a significantly larger amount of circling behaviour over all training (Fig. S6 F, ANOVA, $F_{1,28} = 59.60$, $P < 0.001$).

Thus, the hypothesis of a cerebellar role in the *global tuning* of spatial behaviour, and more specifically in balancing exploration-exploitation behaviour, accounted for the entire set of experimental data in the MWM [1]. The mean intergroup differences were comparable in simulation and experiments for all behavioural parameters (escape latency: ANOVA, $F_{1,18} = 0.0516$, $P > 0.5$; heading: ANOVA, $F_{1,18} = 2.987$, $P > 0.1$; ratio between time spent in the platform quadrant and trial duration: ANOVA, $F_{1,18} = 0.00005$, $P > 0.5$; distance to the platform: ANOVA,$F_{1,18} = 1.59$, $P > 0.1$: and circling: ANOVA, $F_{1,18} = 0.53$, $P > 0.25$). We checked that the difference in circling time between mutants and controls was not sufficient alone to capture all spatial navigation impairments of L7-PKCI mice observed experimentally (not shown).

In the Starmaze task, consistently with experimental data, we did not observe any significant difference between controls and mutants in terms of either mean number of alleys visited (Fig. S6 G, ANOVA $F_{1,18} = 0.32$, $P > 0.5$), or total distance swum (Fig. S6 H, ANOVA $F_{1,18} = 0.45$, $P > 0.5$).

References

1. Burguière E, Arleo A, Hojjati M, Elgersma Y, De Zeeuw CI, et al. (2005) Spatial navigation impairment in mice lacking cerebellar LTD: a motor adaptation deficit? Nat Neurosci 8: 1292–1294.
